# Single‐cell landscape of the cellular microenvironment in three different colonic polyp subtypes in children

**DOI:** 10.1002/ctm2.1535

**Published:** 2024-01-24

**Authors:** Yafei Deng, Canlin Li, Lanlan Huang, Peiwen Xiong, Yana Li, Yongjie Liu, Songyang Li, Weijian Chen, Qiang Yin, Yong Li, Qinglan Yang, Hongyan Peng, Shuting Wu, Xiangyu Wang, Qin Tong, Hongjuan Ouyang, Die Hu, Xinjia Liu, Liping Li, Jieyu You, Zhiyi Sun, Xiulan Lu, Zhenghui Xiao, Youcai Deng, Hongmei Zhao

**Affiliations:** ^1^ Pediatrics Research Institute of Hunan Province and Hunan Provincial Key Laboratory of Children's Emergency Medicine Hunan Children's Hospital Changsha China; ^2^ The School of Pediatrics Hengyang Medical School University of South China Changsha China; ^3^ Department of Digestive Nutrition Hunan Children's Hospital Changsha China; ^4^ Department of Pathology Hunan Children's Hospital Changsha China; ^5^ Department of Pediatric Surgery Hunan Children's Hospital Changsha China; ^6^ Department of Clinical Hematology College of Pharmacy and Laboratory Medicine Science Army Medical University Chongqing China; ^7^ Department of Biostatistics School of Public Health University of Michigan Ann Arbor Michigan USA

**Keywords:** cellular microenvironment, epithelial–mesenchymal transition, paediatric colonic polyps, scRNA‐seq

## Abstract

**Background:**

The understanding of the heterogeneous cellular microenvironment of colonic polyps in paediatric patients with solitary juvenile polyps (SJPs), polyposis syndrome (PJS) and Peutz–Jeghers syndrome (PJS) remains limited.

**Methods:**

We conducted single‐cell RNA sequencing and multiplexed immunohistochemistry (mIHC) analyses on both normal colonic tissue and different types of colonic polyps obtained from paediatric patients.

**Results:**

We identified both shared and disease‐specific cell subsets and expression patterns that played important roles in shaping the unique cellular microenvironments observed in each polyp subtype. As such, increased myeloid, endothelial and epithelial cells were the most prominent features of SJP, JPS and PJS polyps, respectively. Noticeably, memory B cells were increased, and a cluster of epithelial–mesenchymal transition (EMT)‐like colonocytes existed across all polyp subtypes. Abundant neutrophil infiltration was observed in SJP polyps, while CX3CR1^hi^ CD8^+^ T cells and regulatory T cells (Tregs) were predominant in SJP and JPS polyps, while GZMA^hi^ natural killer T cells were predominant in PJS polyps. Compared with normal colonic tissues, myeloid cells exhibited specific induction of genes involved in chemotaxis and interferon‐related pathways in SJP polyps, whereas fibroblasts in JPS polyps had upregulation of myofiber‐associated genes and epithelial cells in PJS polyps exhibited induction of a series of nutrient absorption‐related genes. In addition, the TNF‐α response was uniformly upregulated in most cell subsets across all polyp subtypes, while endothelial cells and fibroblasts separately showed upregulated cell adhesion and EMT signalling in SJP and JPS polyps. Cell–cell interaction network analysis showed markedly enhanced intercellular communication, such as TNF, VEGF, CXCL and collagen signalling networks, among most cell subsets in polyps, especially SJP and JPS polyps.

**Conclusion:**

These findings strengthen our understanding of the heterogeneous cellular microenvironment of polyp subtypes and identify potential therapeutic approaches to reduce the recurrence of polyps in children.

## Graphical abstract

Single‐cell profiling of colonic polyps from pediatric patients with SJP, JPS or PJS.

Our results systematically provide a detailed atlas of the cellular microenvironments of SJP, JPS, and PJS polyps in pediatric patients.

This study provides a valuable resource which can facilitate exploration of the pathogenesis of colonic polyps and identify novel therapeutic targets in the future

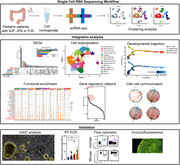

## 1 INTRODUCTION

Juvenile polyps, which constitute 80% of all colonic polyps, are the most frequent type in children.^1^ Although most juvenile polyps have a negligible risk for malignant transformation, some paediatric patients may suffer from anaemia, hypoalbuminemia, diarrhoea and abdominal pain due to intussusception or obstruction caused by the polyps or even prolapse of pedunculated polyps from the rectum.^2^ Juvenile polyps are usually classified into solitary juvenile polyps (SJPs), which show a very low incidence of adenocarcinoma, and polyposis syndromes, which present as either hamartomatous or adenomatous polyps.^1^ The SJPs that are observed in 1% of all preschoolers are also known as inflammatory and cystic polyps, which result from the structural rearrangement of the mucosa caused by inflammation.^1^, ^3^ The hamartomatous polyposis syndromes include juvenile polyposis syndrome (JPS) and Peutz–Jeghers syndrome (PJS). Most children diagnosed with JPS who have more than five colonic polyps have an increased risk of cancer in adulthood, whereas PJS is a rare polyposis syndrome (1/50 000−200 000) associated with a high lifetime risk of gastrointestinal and extraintestinal cancers (37–93%).^3^ Germline mutations in *SMAD4* and *BMPR1A* are reported in 40–60% of children with JPS, while PJS is most often due to a germline mutation in the *STK11/LKB1* genes.^3^ Currently, endoscopic or surgical removal of polyps is often applied to these patients in clinical^4^, ^5^; however, repetitive removal of polyps requires high‐frequency anaesthesia and brings a high risk of enterobrosis for patients with JPS and PJS polyps. Thus, a deep comprehensive understanding of how different polyp subtypes develop within the local microenvironment could reveal potentially valuable new potential preventive or therapeutic targets for different juvenile polyps.

Previous studies have applied immunohistochemistry (IHC) and confocal laser endomicroscopy to characterize the phenotype of the microenvironment in colonic polyps and identified the histological types of polyps (adenomatous or non‑adenomatous) and the degree of dysplasia.^6^, ^7^ These differences not only reflect the high heterogeneity of the microenvironment among different polyp subtypes but also indicate the need for accurate diagnosis and personalized treatment. Although previous reports have described increased inflammatory cell infiltration in SJP polyps^3^ and the transformation of normal epithelium into PJS polyps.^8^, ^9^ To date, an integrated scenario describing the cellular and molecular features of different polyp subtypes at the single‐cell level is still absent.

To explore the microenvironment in polyps and then understand the differences in clinical symptoms and treatments underlying different polyp subtypes, this study mapped the cellular landscape of colonic polyps from paediatric patients with SJP, JPS or PJS and aged‐matched colonic tissues by using single‐cell RNA sequencing (scRNA‐seq) technology to define detailed characterization of different cell subsets and their crosstalk within the microenvironments.^10^, ^11^, ^12^ This study will help us to assist in analysing the heterogeneity of polyps across distinct subtypes and to pinpoint the fundamental cellular and molecular mechanisms that could influence disease prognosis.

## 2 RESULTS

### 2.1 Colonic polyps exhibit disease‐associated shifts in the cellular microenvironment in paediatric patients

**FIGURE 1:**
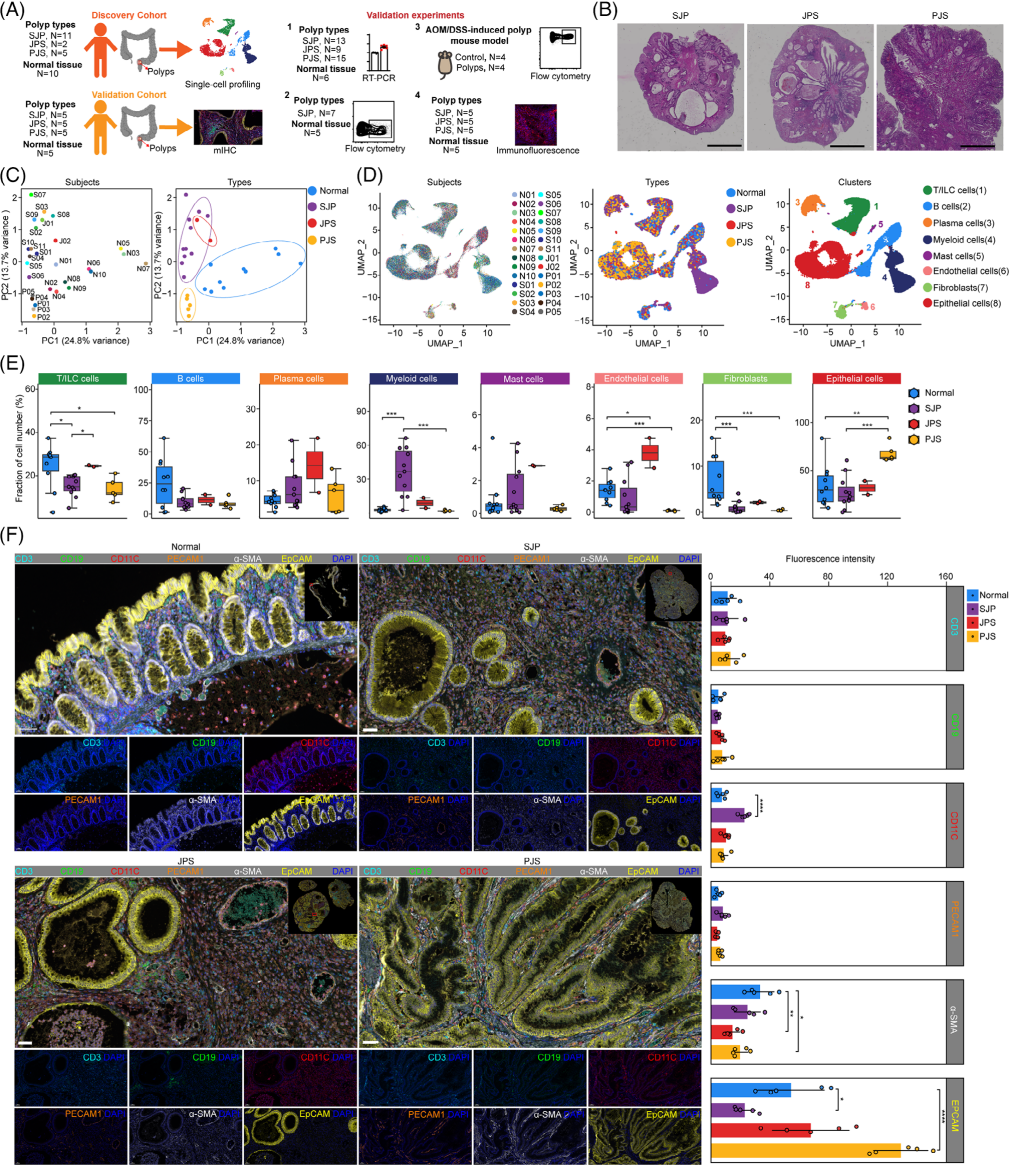
Colonic polyps exhibit disease‐associated shifts in the cellular microenvironment in paediatric patients. (A) An experimental scheme diagram of the overall study design. (B) Immunohistochemical staining identifying typical morphological features of different polyp subtypes. Scale bar, 3 mm. (C) Principal component analysis (PCA) of all 28 samples based on the mean expression of 2000 variable genes for each sample coloured by subject (left) and different sample types (right). (D) UMAP plot of cells from different subjects (left), sample types (middle) and clusters (right). (E) Boxplots comparing the fractions of major cell subsets in different sample types. Statistical analysis was performed using the Kruskal–Wallis test; *p < .05; **p < .01; ***p < .001. (F) Representative mIHC images (left) show staining for CD3 (T cells), CD19 (B cells), CD11C (myeloid cells), PECAM1 (endothelial cells), α‐SMA (fibroblasts) and EpCAM (epithelial cells) in normal tissues and three different polyp subtypes. Scale bar, 50 µm. Quantitation of fluorescence intensity (right) of the indicated antibodies in normal tissues and three different polyp subtypes using ImageJ software. Statistical analysis was performed using the Kruskal–Wallis test; *p < .05; **p < .01; ****p < .0001.

To discover the divergent cellular compositions of different polyp subtypes in paediatric patients, we performed scRNA‐seq of dissociated fresh polyp tissues from 18 patients who underwent colonoscopy or radical surgeries and two normal colon tissues from a colostomy and soave procedure (discovery cohort). Among these 18 polyp samples, 11 SJP samples were from paediatric patients with 1–2 polyps; two typical JPS samples were from two patients who underwent 8 and 14 colonoscopies, respectively, and 112 and 116 polyps were removed during the most recent colonoscopy, respectively; and five PJS samples were from five patients with a germline mutation in the *STK11/LKB1* gene. In addition, eight published scRNA‐seq datasets for normal colon tissues from age‐matched individuals were also integrated as controls.^13^ In addition, we also collected five normal colon tissues as well as 15 polyp tissues (five for each polyp subtype) for multiplexed immunohistochemistry (mIHC) analysis to confirm differences found in the discovery cohort (validation cohort). Moreover, a series of validation experiments, including flow cytometry, a mouse model, RT–PCR and immunofluorescence (IF), were performed (Figure 1A,B and Figure S1A, Table S1).

In the initial quality control process, we observed that some of these samples had over 25% mitochondrial reads, especially PJS polyps. These results might result from that these PJS polyps had relatively high ratios of epithelial cells that were more sensitive to the digestion process. However, these samples showed comparable numbers of unique molecular identifiers (UMIs) and genes per cell with other samples with low mitochondrial reads and no special clinical and experimental information were found that may contribute to the high mitochondrial reads. Thus, we chose a relatively high threshold value (50%) of mitochondrial reads (Table S2). Principal component analysis (PCA) of 2000 variable genes for each sample segregated normal and polyp tissues along PC1 but not by individual, while JPS polyps intermixed with SJP polyps, both of which segregated from PJS polyps along PC2 (Figure 1C). Thus, we think that polyps in children have high heterogeneity of mitochondrial reads which might result from age, sex, diet and other unknown factors.

Uniform manifold approximation and projection (UMAP) embeddings of 116 628 high‐quality cells from these 28 samples showed distinct separation of the major lineages into T/innate lymphoid cell (ILC) cells, B cells, plasma cells, myeloid cells, mast cells, endothelial cells, fibroblasts and epithelial cells (Figure 1D and Figure S1B). Hierarchical clustering analysis based on the expression profiles showed that cell types were clustered together according to the cell type but not the tissue group, except for myeloid cells (Figure S1C). The percentages of cell types were evaluated, and the ratios of T/ILC cells and fibroblasts were reduced in both SJP and PJS but not in JPS polyps, while that of myeloid cells was strongly elevated in SJP but not in JPS or PJS polyps. The level of endothelial cells was specifically increased in JPS polyps, whereas that of epithelial cells was specifically increased in PJS polyps (Figure 1E). However, there were weak correlations of the frequencies of all of these cell types with the polyp weights (Figure S1D). To validate the scRNA‐seq data, a cohort of independent samples was subjected to mIHC, and quantitative analysis of the imaging data showed good agreement with scRNA‐seq, such as increased myeloid cells (CD11C^+^ cells) in SJP polyps and increased epithelial cells (EpCAM^+^ cells) in PJS polyps compared with normal tissues. Moreover, morphologically, PJS polyps typically exhibited epithelial dysplasia with dendritic gland hyperplasia surrounding smooth muscle tissue, whereas SJP and JPS polyps exhibited irregular and enlarged glands (Figure 1F).

Together, these findings strongly reflected a shift in the cellular microenvironment among the three different colonic polyp subtypes.

### 2.2 Shared and unique expression patterns across cell subsets and polyp subtypes in paediatric patients

**FIGURE 2:**
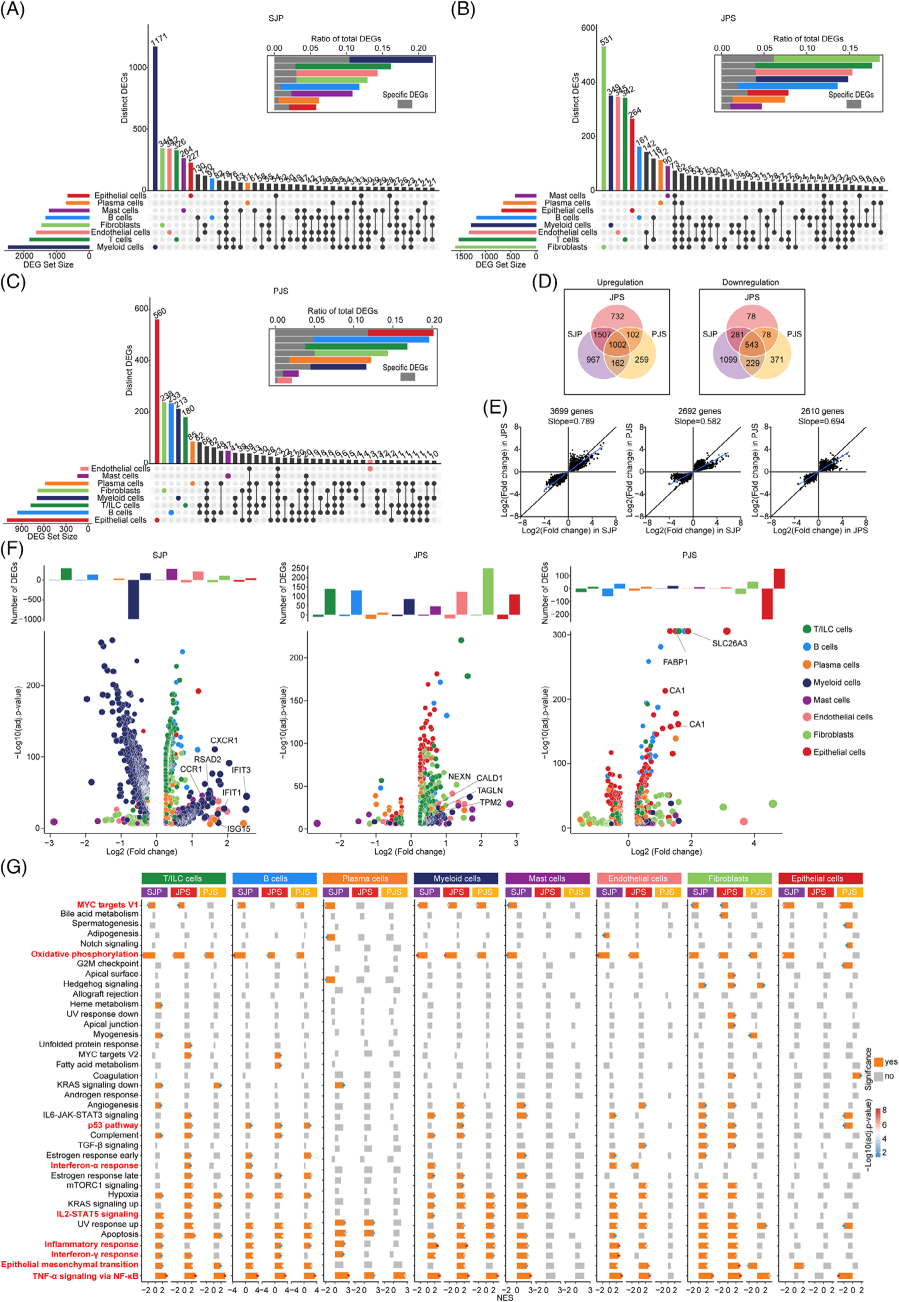
Shared and unique expression patterns across cell subsets and polyp subtypes in paediatric patients. (A–C) Cell‐type specificity of DEGs in SJP (A), JPS (B) and PJS (C) polyps when compared with normal tissues, separately. (D) Venn diagrams of upregulated (left) and downregulated (right) DEGs in SJP, JPS or PJS polyps compared with normal tissue. (E) Distribution of shared DEGs by the given polyp subtypes. (F) Histogram and volcano plot showing the total number (top) and distribution (bottom) of DEGs specific to a cell subset, respectively. (G) Boxplots showing the activities of hallmark gene sets in major lineage subtypes across each polyp type.

We then detected the differentially expressed genes (DEGs) across cell subsets between the different polyp subtypes and normal colon tissues to assess polyp type‐related transcriptomic changes, and the discrepancies were more pronounced in SJP and JPS than PJS polyps in terms of the total numbers of DEGs, indicating that the transcriptional changes in SJP and JPS polyps were, therefore, more marked than those in PJS polyps (Figure 2A–C). SJP, JPS and PJS polyps showed the most DEGs in myeloid cells, fibroblasts and epithelial cells, respectively. The differences in T/ILC cells were uniformly significant in all three polyp subtypes, and the differences in endothelial cells were also significant in SJP and JPS but not PJS polyps, while a small number of DEGs were shared in all cell types among the polyp subtypes. Notably, tremendous numbers of the myeloid cell‐, fibroblast‐ and epithelial cell‐specific DEGs existed in SJP, JPS and PJS polyps, respectively, suggesting that dysregulated myeloid cells, fibroblasts and epithelial cells play critical roles in polyp progression in SJP, JPS and PJS patients, respectively. Besides, in order to investigate the influence of cell number on the number of DEGs, we randomly selected 20%, 50%, 80% and 100% of myeloid cells in SJP polyps and then compared them with myeloid cells in normal tissues, separately. In the same way, we compared different numbers of fibroblasts in normal tissues with fibroblasts in JPS polyps, as well as compared different numbers of epithelial cells in PJS polyps with epithelial cells in normal tissues, separately. The results showed that the vast majority of DEGs were overlapped, which suggested that cell numbers had little influence on the number of DEGs (Figure S2).

Among all these DEGs, 1002 upregulated and 543 downregulated genes were shared across all three polyp subtypes, and SJP had the most unique DEGs, including 967 upregulated and 1099 downregulated genes (Figure 2D). The number of shared DEGs between SJP and JPS polyps was greater than that between SJP and PJS polyps or that between JPS and PJS polyps. Despite significant differences in histological features, SJP and JPS polyps had more similar rangeability of these shared DEGs than JPS and PJS polyps (Figure 2E). Furthermore, plotting these unique DEGs revealed that T/ILC cells, mast cells, endothelial cells and especially myeloid cells were the main type‐specific DEGs in SJP polyps. Myeloid cells exhibited specific induction of genes involved in chemotaxis (*CXCR1* and *CCR1*) and interferon‐related pathways (*IFIT1*, *IFIT3*, *ISG15* and *RSAD2*) in SJP polyps. The type‐specific DEGs in JPS polyps were distributed in all cell subsets except plasma cells, and fibroblasts had the most DEGs, including upregulated myofiber‐associated genes (*NEXN*, *TAGLN*, *TPM2* and *CALD1*). Noticeably, epithelial cells in PJS polyps exhibited induction of a series of genes related to nutrient absorption, such as *FABP1*, *SLC26A3*, *CA1* and *CA4*, which were the dominant type‐specific DEGs in PJS polyps (Figure 2F).

In addition, we also compared the DEGs of these eight main cell subsets between different polyp subtypes and found that myeloid cells in JPS and PJS polyps had more upregulated DEGs than those in SJP polyps, while SJP polyps had more upregulated DEGs in T/ILC cells, B cells, plasma cells, myeloid cells and endothelial cells than JPS polyps and more upregulated DEGs in T/ILC cells, B cells, fibroblasts and epithelial cells than PJS polyps. Meanwhile, when compared with PJS and JPS polyps, PJS polyps had more upregulated DEGs in plasma cells, while JPS polyps had more upregulated DEGs in T/ILC cells, B cells, myeloid cells, fibroblasts and epithelial cells (Figure S3). Furthermore, myeloid cells had increased levels of genes involved in chemotaxis (*CXCL1, CXCL8, CXCL10, CCR1, CXCR1* and *CXCR2*) and genes involved in inflammation (*IL1B* and *TNFSF10*) in SJP polyps compared with JPS or PJS polyps. CXCL signalling is critical for myeloid infiltration,^14^ while *IL1B* and *TNFSF10* are the key genes in TNF‐α signalling and inflammatory response.^15^ Thus, these DEGs in chemotaxis and inflammation might be a response for the abundant myeloid infiltration and myeloid cells had the highest activities of TNF‐α signalling and inflammatory response, respectively. Compared with SJP or PJS polyps, endothelial cells in JPS polyps exhibited increased levels of *DEPP1*, *EDN1*, *CCN1, SELE* or *ACKR1*, which are involved in the regulation of oxidative stress, vascular contraction, cell adhesion or leukocyte chemotaxis. Similar to type‐specific DEGs in PJS polyps, epithelial cells in PJS polyps exhibited increased levels of a series of genes (such as *FABP1*, *SLC26A2*, *SLC26A3*, *CA1* and *CA2*) related to nutrient absorption (Figure S3).

We further investigated the signalling pathways involved in the development of polyps, and the results showed that the TNF‐α response via NF‐κB was uniformly upregulated in all cell subsets of both SJP and JPS polyps (except for epithelial cells) as well as some cell subsets (T/ILC cells, B cells, plasma cells, myeloid cells and fibroblasts) of PJS polyps but downregulated in epithelial cells in PJS polyps (Figure 2G). Additionally, the epithelial–mesenchymal transition (EMT) pathway was significantly upregulated in T/ILC cells and B cells in all three polyp subtypes as well as other cell subsets in one or two polyp subtypes. In addition, other inflammation‐related pathways (e.g. inflammatory response, interferon‐α response, interferon‐γ response and IL2‐STAT5 signalling) were significantly upregulated in some cell subsets of the polyp subtypes, whereas oxidative phosphorylation and MYC targets V1 were significantly downregulated in most cell subsets of all three polyp subtypes. In addition, almost uniquely, the activity of the p53 pathway was downregulated in only epithelial cells of PJS polyps (Figure 2G).

Altogether, these three polyp subtypes had partly shared transcriptional patterns, with the similarity between SJP and JPS polyps being greater than that between JPS and PJS polyps. In addition, SJP exhibited a stronger myeloid‐ and inflammation‐related DEG signature; JPS polyps exhibited more fibroblast‐expressed DEGs, which were involved in myofiber formation; and PJS polyps had distinctive transcriptional changes in epithelial cells, reflecting upregulated nutrient absorption and a downregulated response to TNF‐α as well as decreased p53 activity.

### 2.3 Induction of the TNF‐α response is uniformly observed in polyps in paediatric patients

**FIGURE 3:**
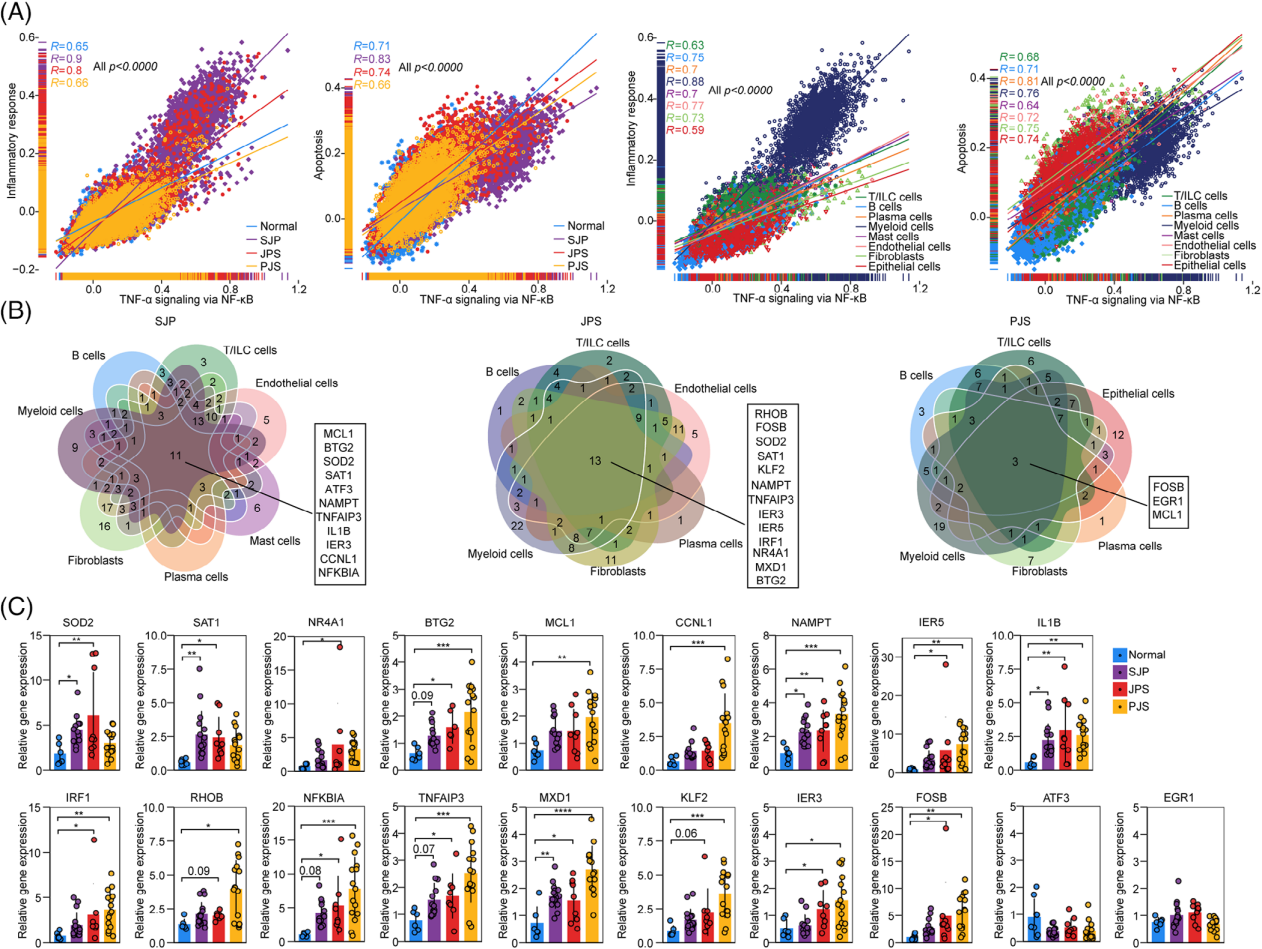
Induction of the TNF‐α response is uniformly observed in polyps in paediatric patients. (A) Scatter plots demonstrating the strong correlations between the activities of TNF‐α signalling via NF‐κB and the inflammatory response or apoptosis across cell subsets and different sample types. (B) Venn diagrams of DEGs involved in TNF‐α signalling via the NF‐κB pathway in given cell subsets of SJP (left), JPS (middle) and PJS (right) polyps. (C) Box and whisker plots of gene expression levels of genes identified in (B) in normal tissues and different polyps, which were detected by RT–PCR. Statistical analysis was performed using two‐sided unpaired Dunn's (Bonferroni) test. *p < .05; **p < .01; ***p < .001; ****p < .0001.

Because pathway enrichment analysis highlighted the TNF‐α response via the NF‐κB pathway in polyps of paediatric patients and activation of the TNF signalling pathway initiates proinflammatory and apoptotic signalling,^16^ the correlations between the activities of the TNF‐α response and the inflammatory response or the apoptotic pathway were evaluated. The results revealed that the activities of the TNF‐α response had strong correlations with the inflammatory response and apoptosis at the levels of both polyp subtype and cell subset (Figure 3A).

Next, we focused on the core DEGs in the gene set of the TNF‐α response via NF‐κB in all cell subsets from each polyp subtype and found that 11, 13 and 3 upregulated DEGs were shared by these cell subsets with a remarkable response to TNF‐α in SJP, JPS and PJS polyps, respectively (Figure 3B). Among these core DEGs, *BTG2*, *SOD2*, *TNFAIP3*, *SAT1*, *NAMPT* and *IER3* were shared by SJP and JPS polyps, while *MCL1* was shared by SJP and PJS polyps. *BTG2* is an antiproliferative gene implicated in cell cycle progression, apoptosis and differentiation.^17^ *TNFAIP3* is rapidly induced by TNF and negatively mediates TNF‐induced NF‐κB activation and apoptosis.^18^ *NAMPT* is a rate‐limiting enzyme in the NAD salvage pathway and is overexpressed in numerous types of cancers, including gastric cancer and colorectal cancer (CRC).^19^ *MCL1*, which is a member of the BCL‐2 family, is overexpressed in many cancers.^20^ All 19 genes were validated by RT–PCR analysis in an independent cohort, and most of these genes, except for *ATF3* and *EGR1*, were upregulated in polyp tissues, especially in PJS polyps (Figure 3C). Thus, these results suggest that increased TNF‐α responses are a common characteristic of polyps in paediatric patients.

### 2.4 Abundant neutrophil infiltration specifically occurs in SJP from paediatric patients

**FIGURE 4:**
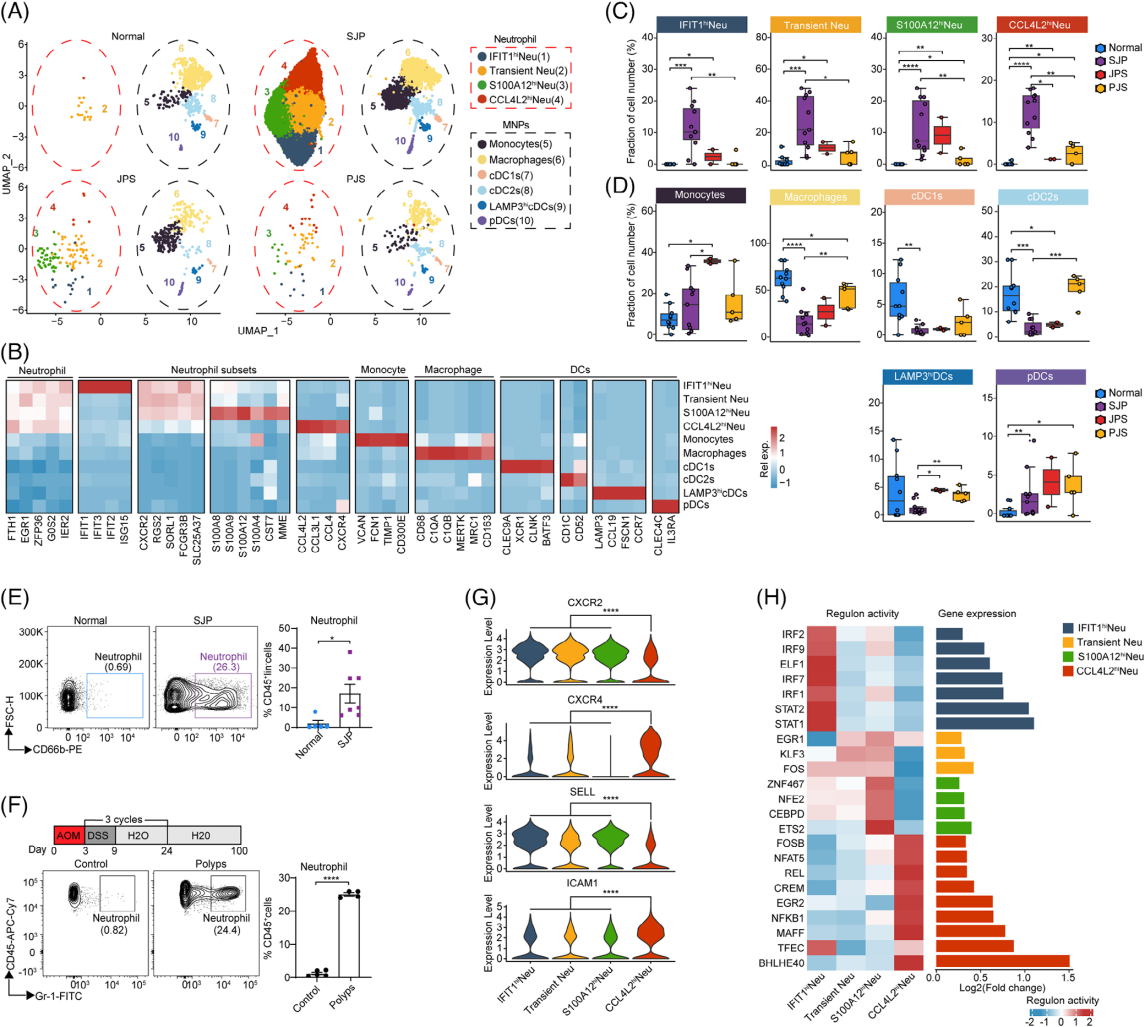
Abundant neutrophil infiltration specifically occurs in SJP from paediatric patients. (A) UMAP plot displaying myeloid cells separated into 10 subsets in different sample types. (B) Heatmap showing subset‐specific marker genes. (C) Boxplots comparing the fractions of neutrophil subsets among myeloid cells in different sample types. Statistical analysis was performed using the Kruskal–Wallis test; *p < .05; **p < .01; ***p < .001; ****p < .0001. (D) Boxplots comparing the fractions of cell subsets among mononuclear phagocytes (MNPs) in different sample types. Statistical analysis was performed using the Kruskal–Wallis test; *p < .05; **p < .01; ***p < .001; ****p < .0001. (E) Flow cytometry demonstrating abundant CD45^+^lin^−^CD66b^+^ neutrophil infiltration in SJP polyps in paediatric patients. Statistical analysis was performed using an unpaired *t* test; *p < .05. (F) The method used for the AOM/DSS‐induced polyp mouse model (top) and flow cytometry results demonstrating CD45^+^Gr‐1^+^ neutrophil infiltration in AOM/DSS‐induced polyps in mice (bottom). Statistical analysis was performed using an unpaired *t* test; ****p < .0001. (G) Vlnplot showing the expression levels of *CXCR2*, *CXCR4*, *SELL* and *ICAM1* in four neutrophil subsets. Statistical analysis was performed using two‐sided unpaired Dunn's (Bonferroni) test; ****p < .0001. (H) Transcription factors inferred by SCENIC. The regulon activity of transcription factors (left) and their fold change in expression levels between the given neutrophil subset and the remaining subsets (right).

As the proportions and activities of the TNF‐α response in myeloid cells were the most significantly increased in polyps, especially SJP (Figures 1E and 3A,B), we further divided myeloid cells into 12 subsets based on cluster‐specific marker genes and previously defined markers^21^ (Figure 4A,B). Strikingly, compared with JPS and PJS polyps, SJP polyps showed marked increases in the proportions of these four neutrophil subsets, while they scarcely existed in normal tissues (Figure 4C,D). The abundant neutrophil (CD45^+^lin^−^CD66b^+^ cell) infiltration in SJP polyps was confirmed by flow cytometric analysis (Figure 4E). Moreover, by establishing a mouse model of azoxymethane (AOM)/dextran sulphate sodium (DSS)‐induced polyps, we found that neutrophil (CD45^+^Gr‐1^+^ cell) infiltration was also markedly increased in colonic polyps compared with normal colon tissue (Figure 4F). This finding is consistent with previous findings that neutrophils are almost absent in normal tissue; however, they can rapidly migrate from peripheral circulating blood to infected tissues in response to inflammatory stimuli.^22^ Moreover, no neutrophil infiltration was reported in polyps from adult patients with familial adenomatous polyposis (FAP)^10^; thus, abundant neutrophil infiltration seems to be a unique characteristic of SJP polyps in children.

Generally, considered short‐lived cells, neutrophils also undergo an ageing process, and these aged neutrophils exhibit proinflammatory phenotypes, accompanied by CXCR4 and ICAM1 upregulation and CD62L (coded by *SELL*) downregulation, promoting their clearance from the circulation and migration to the bone marrow.^23^ Compared with the other three subsets, CCL4L2^hi^ Neu cells had lower levels of *CXCR2* and *SELL* and higher levels of *CXCR4* and *ICAM1*, indicating that CCL4L2^hi^ Neu cells represented a senescent phenotype (Figure 4G). Analysis of the regulons of transcription factors revealed high activity of BHLHE40 in CCL4L2^hi^ Neu cells (Figure 4F). A recent study showed that BHLHE40 was the key transcription factor in the terminal differentiation of neutrophils downstream of hypoxia and endoplasmic reticulum stress.^24^ We also confirmed that CCL4L2^hi^ Neu cells were the most differentiated among these four neutrophil subsets (Figure S4A). The genes with dynamic expression along the trajectory were divided into three clusters, which visualized distinct biological processes associated with neutrophil differentiation. The late upregulated genes, which were mainly distributed in CCL4L2^hi^ Neu, were enriched for the cytokine‐mediated signalling pathway, intrinsic apoptotic signalling pathway, response to hypoxia and response to tumour necrosis factor (Figure S4B). CCL4L2^hi^ Neu cells also had the lowest level of antiapoptotic *MCL1* among these neutrophil subsets (Figure S4C). Furthermore, the late upregulated genes along the trajectory were enriched for the glycolytic process, which agreed with the use of glycolysis for energy production in apoptotic neutrophils.^25^ Moreover, among these four subsets, S100A12^hi^ Neu cells generally had higher extravasation, degranulation, chemotaxis and neutrophil extracellular trap release activities, while CCL4L2^hi^ Neu cells generally had higher reactive oxygen species (ROS) production and angiogenesis activities (Figure S4D).

Collectively, our results present a comprehensive picture of the four neutrophil subsets and clarify that neutrophil infiltration may contribute to the inflammatory phenotype of SJP polyps.

### 2.5 Endothelial cells and fibroblasts separately show upregulated cell adhesion and EMT signalling in SJP and JPS polyps

As the main stromal compartments, endothelial cells and fibroblasts had increased activities of inflammation, immune response‐associated pathways and EMT in SJP and JPS but not PJS polyps (Figure 2G). We extracted endothelial cells and found that these cells in SJP and JPS polyps were remarkably distinct from those in normal tissue, exhibiting high expression levels of *SELE*, *C2CD4B*, *CCN1* and *ICAM1*, all of which are involved in the regulation of vascular permeability, cell adhesion and leukocyte accumulation (Figure S5A,B). Gene Ontology (GO) analysis also revealed that a series of cell adhesion‐associated pathways in the top 10 pathways were upregulated in SJP and JPS but not PJS polyps (Figure S5C).

Fibroblasts in SJP and JPS polyps were also remarkably distinct from those in normal tissue (Figure S5D). These cells highly expressed inflammation‐related genes, including *ATF3*, *IL1R1*, *NFKBIZ* and *WNT5A*, in polyps, especially SJP and JPS polyps (Figure S5E). Specifically, fibroblasts in JPS polyps had increased wingless‐related integration site (WNT) signalling pathway activity and cell–cell signalling by WNT, which are involved in fibrotic progression and EMT in CRC^26^ (Figure S5F).

All these findings suggest that endothelial cells and fibroblasts separately show upregulated activities of cell adhesion and EMT signalling in SJP and JPS polyps.

### 2.6 Memory B cells are increased across different polyp subtypes in paediatric patients

Considering that B cells are heterogeneous, we subclustered B cells into three classical subsets (naive B, memory B and plasmablasts) according to the expression of well‐known marker genes.^11^ (Figure S6A,B). Compared with normal tissues, the percentages of naive B cells were all decreased in these three polyp subtypes, whereas memory B cells were the opposite, which indicated that naive B cells were constantly stimulated by antigens and then differentiated into memory B cells in the polyps. The plasmablasts were not significantly changed (Figure S6C). GO analysis showed that these memory B cells had increased leukocyte cell–cell adhesion activity in SJP and JPS polyps as well as increased T‐cell activity in JPS polyps (Figure S6D).

These results suggest that B cells were activated and that memory B cells were involved in the activation of other immune cells in polyps.

### 2.7 T/ILC cell subsets vary across different polyp subtypes in paediatric patients

**FIGURE 5:**
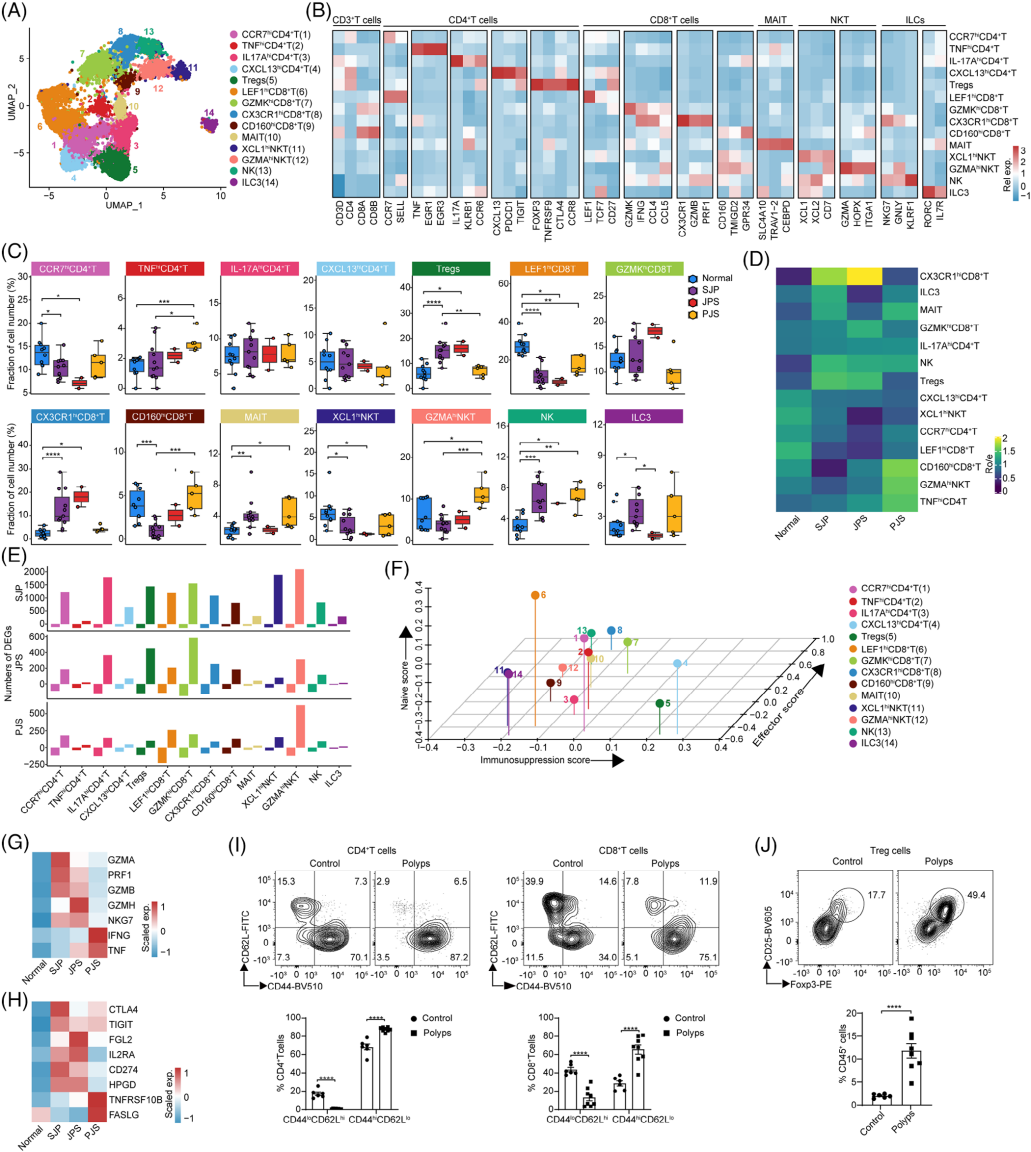
T/ILC cell subsets are vary in different polyp subtypes in paediatric patients. (A) UMAP plot displaying T/ILC cells separated into 14 subsets in different sample types. (B) Heatmap showing subset‐specific marker genes. (C) Boxplots comparing the fractions of T/ILC subsets in different sample types. Statistical analysis was performed using the Kruskal–Wallis test; *p < .05; **p < .01; ***p < .001. (D) Heatmap showing the ORs of T/ILC subsets occurring in different sample types. (E) Boxplots showing the number of DEGs in each T/ILC subset across different polyp subtypes. (F) 3D scatter plot illustrating three feature scores for all T/ILC subsets. (G and H) Heatmap showing the scaled average expression of effector‐related genes in CX3CR1^hi^CD8^+^ T cells (G) and immunosuppression‐related genes in Tregs (H) across normal tissue and different polyp subtypes. (I and J) Flow cytometry showing the ratios of naive (CD44^lo^CD62L^hi^) and effector (CD44^hi^CD62L^lo^) cells in CD4^+^ T (left) and CD8^+^ T (right) cells (I), as well as Treg (CD45^+^CD3^+^CD4^+^CD25^+^FoxP3^+^) cells (J) in AOM/DSS‐induced polyps in mice. Statistical analysis was performed using an unpaired *t* test; ****p < .0001.

We also subclustered T/ILC cells into 14 subsets based on the expression of canonical T/ILC cell markers^27^ (Figure 5A,B). Compared with normal tissues, these three polyp subtypes showed markedly increased percentages of NK cells, whereas the proportions of CCR7^hi^ CD4^+^ T and LEF^hi^ CD8^+^ T cells were reduced in all three polyp subtypes, although the results for CCR7^hi^ CD4^+^ T cells in PJS polyps were not statistically significant. The proportion of XCL1^hi^ NKT cells was reduced, but those of Tregs and CX3CR1^hi^ CD8^+^ T cells were increased in SJP and JPS but not PJS polyps. In addition, the level of CD160^hi^ CD8^+^ T cells was reduced in SJP polyps only, and the percentages of TNF^hi^ CD4^+^ T and GZMA^hi^ NKT cells were increased in PJS polyps only (Figure 5C). According to the ratios of observed to randomly expected cell numbers (Ro/e),^27^ CX3CR1^hi^ CD8^+^ T cells and Tregs were enriched in SJP and JPS polyps, while CD160^hi^ CD8^+^ T, TNF^hi^ CD4^+^ T and GZMA^hi^ NKT cells appeared to be enriched in PJS polyps (Figure 5D). In addition, SJP and JPS polyps exhibited the majority of DEGs in most subsets except for TNF^hi^ CD4^+^ T cells, MAIT cells and ILC3s, whereas PJS polyps showed the majority of DEGs in GZMA^hi^ NKT cells, suggesting that immune cells have wide functional changes in polyps, especially SJP and JPS polyps (Figure 5E).

The well‐known T‐cell feature score revealed that CCR7^hi^ CD4^+^ T cells and LEF^hi^ CD8^+^ T cells exhibited significantly higher naive scores, that CX3CR1^hi^ CD8^+^ T cells exhibited the highest effector score and that Tregs exhibited the highest immunosuppression score (Figure 5F). CX3CR1^hi^ CD8^+^ T cells exhibited higher expression levels of genes associated with cytotoxicity, including *GZMA*, *GZMB*, *GZMH, PRF1* and *NKG7*, in SJP and JPS polyps and higher expression levels of IFNG and TNF in PJS polyps (Figure 5G). Tregs exhibited higher expression of genes associated with exhaustion, including *CTLA‐4*, *TIGIT* and *CD274*, in SJP and JPS polyps and higher expression of molecules, including *FASLG* and *TNFRSF10B*, in PJS polyps (Figure 5H). Moreover, we found that both CD4^+^ T and CD8^+^ T cells exhibited an activated state, as evidenced by decreased ratios of naive (CD44^lo^CD62L^hi^) and increased effector (CD44^hi^CD62L^lo^) cells. Meanwhile, the ratio of Treg cells was also increased in AOM/DSS‐induced polyps compared with normal tissues (Figure 5I,J).

Collectively, these data suggest that both the ratio and activation of CX3CR1^hi^ CD8^+^ effector T cells and immunosuppressive Tregs were upregulated in SJP and JPS polyps, whereas GZMA^hi^ NKT cells were predominant in PJS polyps.

### 2.8 A cluster of EMT‐like colonocytes among polyps in paediatric patients

Although the initial cell type of colonic polyps is a matter of debate, the transformation of the normal epithelium into polyps is unquestionable.^8^, ^9^ We extracted epithelial cells and subclustered them into eight subsets according to previously defined marker genes^28^ (Figure S7A,B). Previous studies have shown that the crypt‐axis score determined using 15 epithelial cell‐expressed genes could in silico‐localize cells within the colonic crypt based on gene expression gradients between single epithelial cells, consistent with an axis of differentiation that ascends through the crypt.^13^, ^28^ Indeed, LGR5^+^ stem cells, which localized to the crypt bottom, had the lowest crypt‐axis score, and colonocytes had the highest score, which indicated a differentiated state (Figure S7C). The proportions of LGR5^+^ stem cells and colonocytes were decreased in polyps compared with normal tissue, while those of early colonocytes and goblet cells were increased in all polyp subtypes, although some polyp subtypes did not have statistically significant results. PJS polyps specifically showed significantly increased levels of BEST4^+^ colonocytes and goblet cells (Figure S7D).

DEG analysis revealed that many DEGs existed in colonocytes and early colonocytes in all three polyp subtypes (Figure S7E). Furthermore, gene set enrichment analysis (GSEA) revealed upregulation of the tumorigenesis‐related pathway EMT in early colonocytes and colonocytes in all three polyp subtypes, although the difference was not significant in PJS polyps, while universal downregulation of cell cycle‐related pathways, including MYC targets V1, MYC targets V2, E2F targets and G2M checkpoint, was observed in early colonocytes and/or colonocytes across all three polyp subtypes, especially PJS. Moreover, the p53 pathway was downregulated in only early colonocytes and colonocytes in PJS polyps (Figure S7F).

**FIGURE 6:**
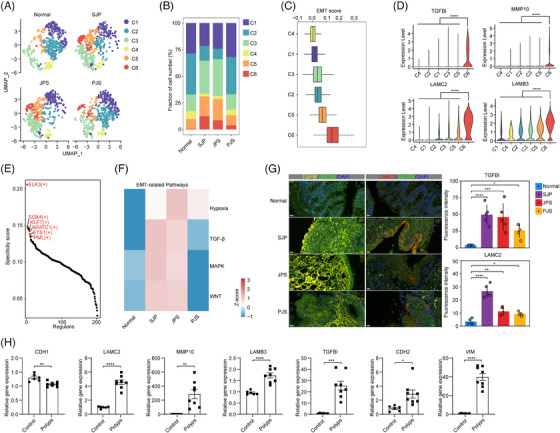
A cluster of EMT‐like colonocytes within polyps in paediatric patients. (A) UMAP plot displaying colonocytes separated into six subsets in different sample types. (B) Bar plot showing the cellular proportions of colonocyte subsets across different sample types. (C) Box and whisker plots of the EMT score. (D) Vlnplot showing the expression levels of *TGFBI*, *MMP10, LAMC2* and *LAMB3* in colonocyte subsets. Statistical analysis was performed using two‐sided unpaired Dunn's (Bonferroni) test. ****p < .0001. (E) Scatter plot displaying the rank of specificity of regulon activity of the C6 subset of colonocytes. The EMT‐related TFs in the top 10 are labelled (red). (F) Heatmap showing the activities of EMT‐related pathways in the C6 subset of colonocytes across different sample types. (G) Immunofluorescence staining (left) showing TGFBI and LAMC2 expression in different sample types. Scale bar, 20 µm. Quantitation of fluorescence intensity (right) of the indicated antibodies in normal tissues and three different polyp subtypes using ImageJ software. Statistical analysis was performed using the Kruskal–Wallis test; *p < .05; **p < .01; ***p < .001; ****p < .0001. (H) Box and whisker plots of gene expression levels of genes in AOM/DSS‐induced polyps in mice, which were detected by RT–PCR. Statistical analysis was performed using an unpaired *t* test; *p < .05; **p < .01; ***p < .001; ****p < .0001.

EMT is a cellular process in which epithelial cells acquire mesenchymal phenotypes and behaviours.^29^ The upregulation of the EMT pathway prompted us to further extract and subcluster colonocytes into six subsets, and the C6 subset was found in all three polyp subtypes but not in normal tissue (Figure 6A,B). Moreover, the C6 subset had the highest score for EMT accompanied by high expression levels of TGF‐β‐induced (*TGFBI*), implicating the classic EMT regulators *TGF‐β*, *MMP10*, *LAMC2* and *LAMB3*, which are typical genes in EMT^30^ (Figure 6C,D). In addition, the transcriptional regulatory network revealed that the top 10 regulons of the C6 subset comprised a series of EMT‐related transcription factors, including ELK3, SOX4, KLF7, NFATC1, ETS1 and PML^31^, ^32^ (Figure 6E). These results indicated that the C6 subset represented an EMT‐like phenotype. In contrast to this EMT‐like phenotype in polyps of paediatric patients, epithelial cells exhibited stem‐like phenotypes during the transformation from normal tissue to polyps in adult patients.^10^ Interestingly, compared with normal tissue, SJP and JPS polyps showed upregulation of the pathways involved in EMT signalling, including hypoxia, TGF‐β, MAPK and WNT, in the C6 subset, while only hypoxia and TGF‐β were upregulated in PJS polyps (Figure 6F). In addition, the activities of these EMT‐related pathways in the C6 subset of JPS polyps were also higher than those of PJS polyps. Finally, through IF staining, we also revealed increased levels of TGFBI and LAMC2 in all three polyp subtypes (Figure 6G). Compared with normal tissues, the mRNA expression level of the epithelial cell marker *Cdh1* (coding E‐cadherin) was decreased, while the levels of EMT‐related genes, including *Tgfbi, Mmp10*, *Lamc2, Lamb3, Cdh2* (coding N‐cadherin) and *Vim* (coding vimentin), were significantly increased in AOM/DSS‐induced polyps (Figure 6H).

All these data revealed that early colonocytes and colonocytes showed decreased cell cycle‐related pathway activity across all polyp subtypes and a downregulated p53 pathway in PJS polyps. Moreover, some colonocytes were transformed into an EMT‐like phenotype among all three polyp subtypes in paediatric patients, with higher EMT signalling in this cluster in both SJP and JPS polyps.

### 2.9 Ignition of an intercellular network within polyps in paediatric patients

**FIGURE 7:**
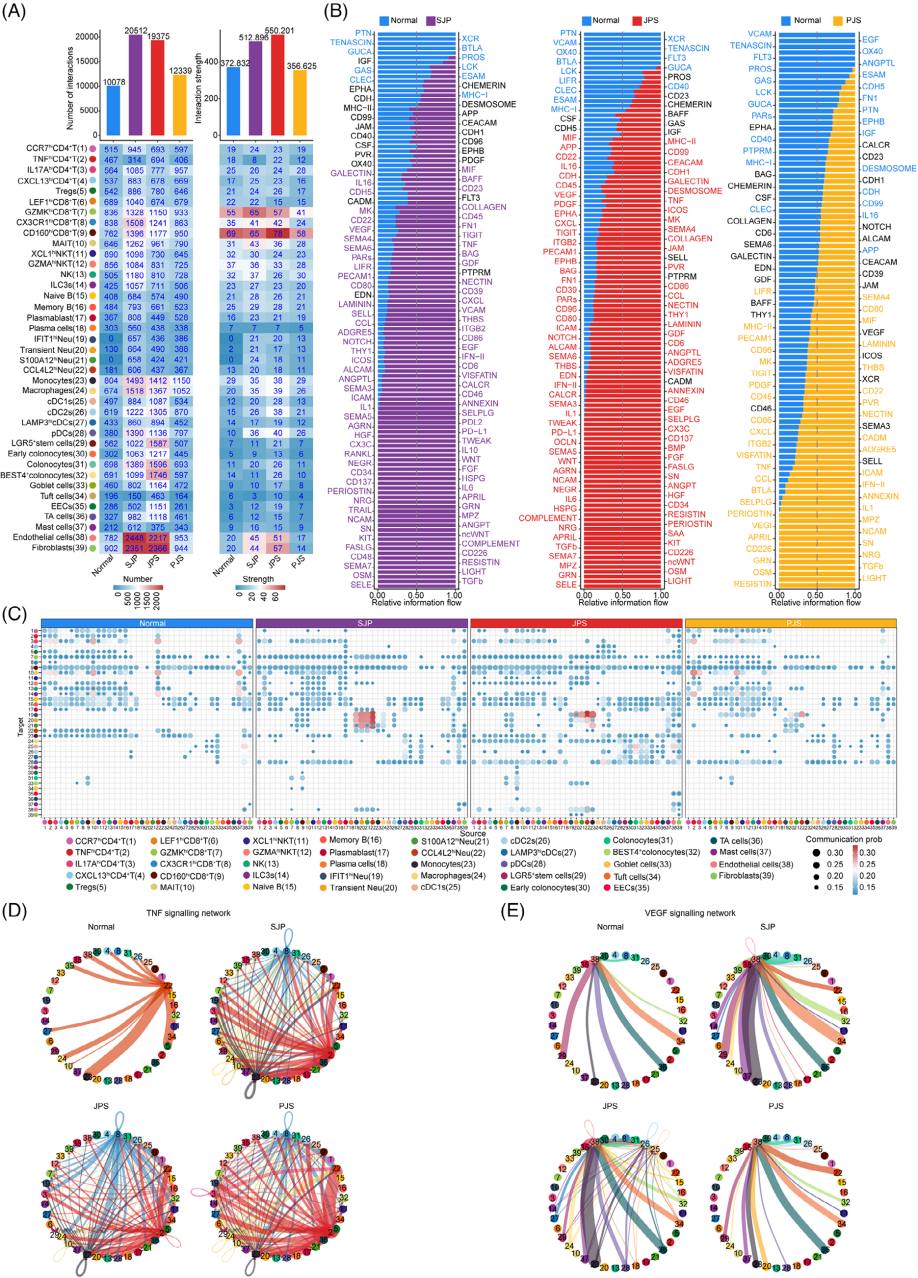
Ignition of an intercellular network within polyps in paediatric patients. (A) Bar plot (top) showing the total number and strength of ligand–receptor interactions and the matrix layout (bottom) of the number and strength of each cell subset across different sample types. (B) Relative information flow for each signalling pathway across different sample types. (C) Dot plot displaying the communication probability among cell subsets across different sample types. (D and E) Circle plot displaying the interactions of the TNF (D) and VEGF (E) signals among cell subsets across normal tissue and different polyp subtypes.

To chart the remodelling of cell–cell interactions in polyps more generally, we mapped interaction pairs onto cell subsets to construct a putative cell–cell interaction network.^33^ Overall, the total number and strength of interactions in SJP and JPS polyps were increased, reflecting enhanced intercellular communication, whereas PJS polyps had a slightly increased number but decreased strength of interactions (Figure 7A). The relative information flow for each signalling pathway was evaluated, and a large proportion of pathways (e.g. TNF, TGF‐β, ICOS and CXZCL) were greatly enhanced, while some pathways (e.g. CD23, MHC‐I, CLEC and TENASCIN) were attenuated in all three polyp subtypes (Figure 7B).

To further depict the remodelling of cell–cell interactions, we mapped the communication probability of cell–cell interactions across polyps, and the speculative results showed that communication among four neutrophil subsets in SJP polyps was more probable than that among other cell subsets. In addition, epithelial cells had increased interactions with monocytes, macrophages and three dendritic cell (DC) subsets, while fibroblasts had increased interactions with other subsets, including T cells, monocytes and plasmacytoid DCs (pDCs), in SJP and JPS polyps. Furthermore, endothelial cells had increased interactions with neutrophils in SJP and JPS polyps and parts of T/ILC subsets in JPS polyps (Figure 7C).

In agreement with the upregulated TNF signalling in polyps, the TNF signalling network was strongly increased in all three polyp subtypes. Most cell subsets are involved in TNF signalling, and TNF^hi^ CD4^+^ T and CX3CR1^hi^CD8^+^ T cells were the main sources in polyps (Figure 7D). Further analysis suggested that compared with normal tissues, TNF^hi^ CD4^+^ T and CX3CR1^hi^CD8^+^ T cells had increased expression levels of *TNF*, while many cell subsets, such as IL‐17A^+^CD4T, Tregs, four neutrophil subsets, monocytes, macrophages and three DC subsets, also had increased expression levels of TNF receptors (*TNFRSF1A* and *TNFRSF1B*), especially *TNFRSF1B* (Figure S8B). In addition, the VEGF signalling network acting on endothelial cells was increased in SJP and JPS polyps, reflecting that monocytes, pDCs and epithelial subsets, including LGR5^+^ stem cells, tuft cells and TA cells, were the main cell subsets interacting with endothelial cells in normal tissue, while mast cells and/or monocytes were the main cell subsets in polyps, which agreed with the increased activity of endothelial cell adhesion in SJP and JPS polyps (Figure 7E). Specifically, compared with normal tissues, monocytes, pDCs and epithelial subsets had increased expression levels of *VEGFA* and/or *VEGFB*, while endothelial cells had increased expression levels of VEGF receptors *FLT1* and *KDR* in three polyp subtypes (Figure S8C).

Chemokines are crucial for orchestrating neutrophil migration.^14^ CXCL signalling, including the CXCL8‐CXCR2 and CXCL8‐CXCR1 pairs, mainly existed among the neutrophil subsets themselves and was increased in all three polyp subtypes compared with normal tissue (Figures S8A and S9A). These results suggest that autocrine action among neutrophil subsets promotes the infiltration of neutrophils into polyps, especially JPS polyps, through CXCL signalling. In addition, monocytes and macrophages also interacted with neutrophil subsets by enhancing CXCL2‐CXCR2 and CXCL3‐CXCR2 pairs to promote neutrophil migration (Figures S8A and S9C). The collagen signalling network by fibroblasts was also increased, which was consistent with the obvious increase in EMT signalling in fibroblasts in both SJP and JPS but not PJS polyps (Figure S9B). Within the collagen signalling network, fibroblasts acted on most of the cell subsets except for tuft cells and endothelial cells through the COLIA1/COLIA2/COL6A3‐CD44 pairs (Figure S8A). Specifically, compared with normal tissues, fibroblasts had increased expression levels of *COLIA1*, *COLIA2* and *COL6A3* in polyps, especially SJP and JPS polyps, and most cell subsets had increased expression levels of *CD44* (Figure S9D).

Overall, intercellular communication among most cell subsets, including neutrophil subsets, endothelial cells and fibroblasts, was markedly enhanced in polyps, especially SJP and JPS polyps. This rewiring of cell–cell interactions may explain the infiltration and activation of cell subsets during polyp progression.

## 3 DISCUSSION

Although rarely malignant, colonic polyps, which are commonly encountered, are one of the most common causes of rectal bleeding and nutritional anaemia during childhood. In this study, we systematically provide a detailed atlas of the cellular microenvironments of SJP, typical JPS and PJS polyps in paediatric patients at single‐cell resolution, identifying type‐specific and type‐shared cell subsets as well as expression patterns for each polyp subtype.

Compared with patients with SJP or JPS polyps, PJS patients had more definitive pathogenesis. All PJS patients participating in this study had germline mutation in the *STK11/LKB1* gene. *STK11/LKB1* is involved in the regulation of cell polarity, proliferation, migration, the DNA damage response and p53‐mediated apoptosis.^3^ Polyp progression is usually accompanied by epithelial dysplasia.^34^ Consistently, our data revealed that the proportion of epithelial cells was specifically increased, up to approximately 60% of total cells, in PJS but not in SJP or JPS polyps. Analysis of changes in pathways in colonocytes revealed that the pathways associated with cell cycle checkpoints were downregulated across all three polyp subtypes, while the p53 pathway and apoptosis were uniquely downregulated in colonocytes of PJS polyps, which suggested that *STK11/LKB1* mutation made colonocytes prone to DNA damage and malignancy in PJS polyps, while p53‐targeted drugs may be used to delay polyp progression in PJS patients. Interestingly, the cell–cell communication network analysis revealed that compared with normal tissues, PJS polyps showed only a slightly increased number of interactions but reduced strength of intercellular communication, which was not similar to the dramatically enhanced intercellular communication in both SJP and JPS polyps. In particular, the activity of the collagen signalling network, which responds to extracellular matrix (ECM) remodelling, was reduced in PJS polyps compared with normal tissues. This phenomenon might be explained by the fact that in PJS patients, intrinsic *STK11/LKB1* mutation results in epithelial dysplasia and then polyp progression, which is different from the increased infiltration of myeloid cells and enhanced inflammatory response in the other two polyp subtypes, especially SJP.

Our analyses also revealed drastic changes in myeloid cells in SJP polyps. In contrast to adult patients with FAP,^10^ we first reported that substantial neutrophil infiltration was the most prominent feature of polyps in paediatric SJP patients, accounting for up to approximately 15% of total cells, compared with the other 2 polyp subtypes. As the high plasticity of neutrophils is reflected by the heterogeneous phenotypes in the tumour microenvironment,^35^ we also identified four neutrophil subsets in SJP polyps, and CCL4L2^hi^ Neu represented a more differentiated and aged phenotype, which had strong activities of TNF‐α signalling, ROS production and angiogenesis. Cell–cell communication analysis indicated that the infiltration of neutrophils was mainly mediated by autocrine action through CXCL signalling and that this positive feedback loop may lead to neutrophil accumulation. All this evidence demonstrates that the infiltration and reprogramming of neutrophils play pivotal roles in proinflammatory processes, oxidative stress and angiogenesis in SJP polyp progression.

In addition, we found that all three polyp subtypes were characterized by upregulated TNF‐α signalling via the NF‐κB pathway, which was also positively correlated with the inflammatory response and apoptosis. Among these eight main cell subsets, myeloid cells had the highest activities of TNF‐α signalling, inflammatory response and apoptosis, which was robust evidence to confirm that SJP was an inflammatory polyp due to abundant myeloid cell (mainly neutrophil) infiltration specifically occurring in the SJP of paediatric patients. The binding of TNF‐α to its receptors (TNFR1 and TNFR2, coded by *TNFRSF1A* and *TNFRSF1B*, respectively) effectively stimulates apoptosis and necrotic cell death, which can lead to inappropriate acute and chronic inflammatory processes, resulting in severe pathologies, such as inflammatory bowel disease.^36^ Cell–cell communication analysis showed that the main source of the TNF signalling network was CCL4L2^hi^ Neu cells in normal tissue but shifted to TNF^hi^ CD4^+^ T cells, and many cell subsets had increased expression levels of *TNFRSF1A* and *TNFRSF1B* in all three polyp types. A previous study showed that mice with T‐cell *STK11/LKB1* deficiency also showed an increased expression level of *Tnf* in gastrointestinal polyps.^9^ All these results suggest that active TNF‐α signalling is a universal phenomenon among polyps, indicating that the use of anti‐TNF therapy might be an alternative strategy to reduce the recurrence of polyps in JPS, PJS and SJP paediatric patients with coagulation disorders or unstable vital signs who are not unsuitable for polypectomy.

A recent study revealed that some epithelial cells exhibited stem‐like phenotypes in polyp and CRC in adult patients,^10^ while our data revealed a common subcluster of EMT‐like colonocytes in all three polyp subtypes. EMT is a cellular process in which epithelial cells acquire mesenchymal phenotypes as well as behaviours and exist in various physiological and pathological conditions.^29^ These EMT‐like colonocytes showed higher expression levels of *TGFBI*, *LAMC2*, *LAMB3* and *MMP10*, which have been known to reduce cell adhesion, promote cell migration and ECM degradation, respectively.^37^, ^38^, ^39^ Moreover, this EMT process was also confirmed by decreased *Cdh1* and increased *Cdh2* and *Vim* levels in a mouse model of AOM/DSS‐induced polyps. Thus, EMT may be an important driver of polyp progression irrespective of polyp subtype. Although both JPS and PJS polyps were hamartomatous, these EMT‐like colonocytes in SJP and JPS polyps had higher activities of EMT‐related pathways than did those in PJS polyps. Consistently, our data revealed that upregulated EMT signalling also existed in endothelial cells and fibroblasts, while epithelial cells exhibited increased interaction with myeloid cells in both SJP and JPS but not PJS polyps. Therefore, we speculated that these drastic immune and inflammatory responses promoted the EMT process and exacerbated polyp progression in paediatric patients with SJP and JPS, while intrinsic *STK11/LKB1* mutation might induce EMT in PJS polyps.

The roles of epithelial, immune and stromal cells and their crosstalk in regulating disease development have been well‐reported.^40^ Here, we identified enhanced cell adhesion by endothelial cells in both SJP and JPS but not PJS polyps. VEGF signalling in endothelial cells is involved in angiogenesis, vascular permeability and microinflammatory cascades, and antiangiogenic drugs have been approved for the treatment of cancer as well as several other disorders.^41^ We found that VEGF signalling was enhanced and/or appeared between endothelial cells and other cell subsets, especially monocytes and mast cells, in SJP and JPS polyps. Similarly, an enhanced proinflammatory response by fibroblasts was also found, and these activated fibroblasts promoted the migration of T/ILCs as well as myeloid cells to polyp tissues by secreting chemokines, such as CXCL2, CXCL3 and CXCL8 and interacting with most other cell subsets through the collagen signalling network in SJP and JPS polyps but not PJS polyps. Within the collagen signalling network, the COL1A1/COL1A2/COL6A3–CD44 interaction pairs existed between fibroblasts and most other cell subsets, especially monocytes but not tuft or endothelial cells. Previous studies have revealed that CD44 participates in cell–cell interactions, cell adhesion and migration, inflammation and the EMT process. Targeting CD44, including neutralizing antibodies or pharmacological inhibitors, is being studied in preclinical and clinical trials for cancer therapy.^42^ These results indicate that therapeutic strategies targeting VEGF or CD44 will also be beneficial for SJP and JPS patients.

Overall, this comprehensive multi‐cellular description dissects heterogeneous cellular microenvironments, laying the foundation for further characterizing the complex and dynamic responses of different polyp subtypes, and thus exploring promising therapeutic strategies to reduce polyp recurrence and malignancy. For patients with JPS polyps, our study identifies several potential approaches, including anti‐TNF, anti‐CD44 and anti‐VEGF therapies, to reduce polyp recurrence after polypectomy or serve as palliative treatments if polypectomy is not feasible. For patients with PJS polyps, we can choose to target p53 signalling to reduce polyp recurrence after polypectomy or employ palliative treatments if polypectomy is not an option.

## 4 METHODS

### 4.1 Paediatric tissue sampling

Colonic polyps were obtained from paediatric patients undergoing colonoscopy or radical surgeries, and normal colonic tissue samples confirmed to be non‐inflamed by pathologists were obtained from age‐matched paediatric patients undergoing a colostomy or soave procedure at Hunan Children's Hospital. The classification of colonic polyps was performed according to clinical symptoms, family history, number of polyps, recurrence and genetic sequencing. Details of the clinical characteristics are summarized in Table S1.

### 4.2 AOM/DSS‐induced polyp mouse model

Approximately 6 week‐old C57BL/6 mice purchased from Hunan Sja Laboratory Animal Co., Ltd. were housed in a specific pathogen‐free facility at Hunan Children's Hospital. The AOM/DSS‐induced polyp formation procedure was carried out according to a previous study protocol.^43^ Briefly, a combination of AOM (MedChemExpress) with repeated treatment with DSS (m.w. 36–50 kDa; MP Biomedicals) in drinking water was used. The mouse was treated with a single intraperitoneal injection of 12 mg/kg AOM. Three days later, 2% DSS dissolved in the drinking water was administered for 6 days, followed by 15 days of regular drinking water. The DSS treatment regimen was repeated for another two cycles. One hundred days after AOM administration, the mouse was sacrificed for further analysis. The control group received one intraperitoneal injection of normal saline and was given unlimited access to normal drinking water.

### 4.3 Paediatric tissue dissociation

For the preparation of single‐cell suspensions from human tissues, both normal colon and polyp tissues were processed using the same protocol. Briefly, following surgical resection, tissues were immediately placed in RPMI 1640 medium (Gibco) supplemented with 5% foetal bovine serum (FBS) (Gibco) and transported on ice to the laboratory. The tissues were minced on ice into < 1 mm^3^ pieces and transferred to Hanks’ solution containing 1 mg/mL collagenase IV (Gibco), 1 mg/mL hyaluronidase (Sigma–Aldrich) and .5 mg/mL DNase I (Sigma–Aldrich) supplemented with 5% FBS for 30 min at 37°C. Next, ice‐cold RPMI 1640 medium with 5% FBS was added. After centrifugation at 450 × *g* and 4°C for 5 min, the supernatant was discarded, and the cell pellet was resuspended in red blood cell lysis buffer. After a 5‐min incubation at room temperature, the samples were centrifuged (450 × *g*, 4°C, 5 min) and then resuspended in phosphate‐buffered saline (PBS) containing .04% BSA. The prepared cells were then counted and assessed for viability with Trypan blue (Corning Inc.) using a blood cell counting chamber. The cells were then resuspended at a concentration of 1000–1500 cells/µL with viability of > 80% for scRNA‐seq.

### 4.4 Mouse tissue dissociation

Single‐cell suspensions from mouse colon tissue and polyps were prepared as previously reported.^44^ Briefly, the colon or polyp tissues were washed with PBS, cut into pieces and gently shaken in D‐Hanks’ solution (pH 7.4) containing 10 mM HEPES, 5 mM EDTA, 1 mM DTT and 10% FBS for 20 min at 37°C. The digests were centrifuged to collect colonic epithelial cells. The remaining tissues were rinsed with Hanks’ solution (pH 7.4) containing 1 mg/mL collagenase II (Gibco) and 10% FBS for 40 min at 37°C. The collected digestion fluid was filtered through a 70‐micron mesh and centrifuged (450 × *g*, room temperature, 10 min) using a 25% Percoll solution (GE Healthcare) in RPMI 1640 medium to concentrate the colon lymphocytes.

### 4.5 Flow cytometry analysis

Standard protocols for flow cytometry were used.^44^ Briefly, the cell suspensions obtained from the normal colon and polyp tissues of patients after tissue dissociation were incubated with anti‐CD45‐PerCP‐Cy5.5 (BioLegend, #304025), anti‐CD3e‐APC‐Cy7 (BD Biosciences, #557832), anti‐CD19‐PE‐Cy7 (BD Biosciences, #557835) and anti‐CD66b‐PE (BioLegend, #392903) antibodies in staining buffer for 15 min at room temperature in the dark. Then, the cells were centrifuged (450 × *g*, 4°C, 5 min) and resuspended in PBS.

Following the same protocol, the cell suspensions of normal colon and polyp tissues of mice were incubated with anti‐CD45‐APC‐Cy7 (BioLegend, #103154) and anti‐Gr‐1‐FITC (BioLegend, #108406) to detect neutrophils. Cell suspensions were incubated with anti‐CD45‐APC‐fire750 (BioLegend, #103154), anti‐CD3‐Alexa Fluor700 (BioLegend, #100216), anti‐CD19‐PE‐Cy7 (BioLegend, #115520), anti‐CD4‐PerCP‐Cy5.5 (BD Biosciences, #550954), anti‐CD8‐BV421 (BD Biosciences, #563898), anti‐CD44‐BV510 (BioLegend, #103044) and anti‐CD62L‐FITC (BioLegend, #104405) to analyse the activation of CD4^+^T and CD8^+^T cells. For Treg cell analysis, cell suspensions were incubated with anti‐CD45‐APC‐fire750, anti‐CD3‐Alexa Fluor700, anti‐CD19‐PE‐Cy7, anti‐CD4‐PerCP‐Cy5.5 and anti‐CD25‐BV605 (BioLegend, #102036), permeabilized with the Foxp3/Transcription Factor Staining Buffer Set Kit (eBioscience) and stained with anti‐Foxp3 (BD Biosciences, #563101) antibody. The BD FACS LSRFortessa instrument (BD Biosciences) was used and data were analysed with FlowJo software (version 10.8.1, FlowJo LLC).

### 4.6 Quantitative RT–PCR analysis

For human and mouse tissues, total RNA was purified with the Total RNA Purification Micro Kit (Norgen Biotek Corp) and reverse transcribed into cDNA using standard protocols as previously described.^45^ Real‐time PCR was performed using the SYBR Green Premix Pro Taq HS qPCR Kit (Accurate Biotechnology (Hunan) Co., Ltd.). The cycle threshold (Ct) values were normalized to those for the internal controls (*GAPDH* for human samples; *β‐actin* for mouse samples). The primer pairs used are shown in Table S3.

### 4.7 Staining of tissue sections

Formalin‐fixed and paraffin‐embedded normal colonic and polyp tissues from paediatric patients sectioned to 4 µm were used for histological evaluation. Haematoxylin and eosin staining was performed on each polyp sample. For IF staining, tissue slides were deparaffinized, rehydrated and processed to antigen retrieval. Primary antibodies for anti‐EpCAM (1:100, Invitrogen, Life Technologies, #14‐9326‐82), anti‐TGFBI (1:150, Invitrogen, #MA5‐32736) and anti‐LAMC2 (1:750, Invitrogen, #PA5‐109901) were used. The slides were then incubated with secondary antibodies for 10 min at room temperature and counterstained for nuclei with DAPI (2 µg/mL, Invitrogen, #D1306) for 15 min. The images were scanned with a laser scanning confocal microscope (Carl Zeiss Meditec AG). For mIHC analysis, primary antibodies against CD3 (1:5, MXB Biotechnologies, #MAB‐0740), CD19 (1:2000, Cell Signaling Technology, #90176), EpCAM (1:2000, Abcam, #ab223582), PECAM1 (1:5, MXB Biotechnologies, #MAB‐0720), CD11C (1:2000, Cell Signaling Technology, #45581) and α‐SMA (1:2000, Boster Biological Technology, #BM0002) were used. Multiplex IF staining was performed using the Opal Polaris 7 Color IHC Detection Kit (Akoya Bioscience, #NEL871001KT), and multispectral images were scanned with PhenoImager HT (Akoya Bioscience).

### 4.8 Droplet‐based scRNA‐seq

Single‐cell suspensions of normal colonic tissues and polyps were loaded within 15 min of completion of cell suspension preparation. Chromium Single‐Cell 3´ reagent kits v3.1 and library kits (10X Genomics) were used and then the libraries were sequenced on an Illumina NovaSeq 6000 (Berry Genomics).

### 4.9 Processing fastq files and quality control

Except for the raw sequencing data from two normal colon tissues and 18 polyps generated by us, the raw sequencing data generated from other normal colon tissues from eight subjects included in this study can be accessed on the EMBL‐EBI database (https://www.ebi.ac.uk/biostudies/arrayexpress) with the accession number E‐MTAB‐8901. All raw sequencing data were processed using CellRanger software v.6.0.0 and the GRCh38 human genome as the reference with other parameters set to default. The cells in each sample were also filtered for less than 50% mitochondrial reads, and all genes that were not detected in ≥3 cells were discarded. Cells with fewer than 200 genes or greater than 5000 total genes, as well as fewer than 1000 UMIs, were also removed. In addition, the R package ‘scDblFinder’ was used to identify doublets.^46^

### 4.10 Data normalization, dimension reduction and batch effect correction

After quality control, the ‘NormalizedData’, ‘ScaleData’ and ‘FindVariableFeatures’ functions in the Seurat package (version 4.3.0) with standard settings were applied for normalization, scaling and variable gene selection, respectively.^47^ To reduce the dimensionality of the data, we ran PCA using the ‘RunPCA’ function with the top 30 principal components, which revealed the main axes of variation and denoised the data. To account for different samples, different datasets and technical sources of variation, we applied a batch effect correction to remove possible batch effects using Harmony.^48^

### 4.11 Unsupervised clustering and annotation

After batch effect correction, Seurat was again used to perform dimensionality reduction and clustering. The neighbourhood graph was based on k = 20 neighbours and the community identification algorithm implemented in the ‘FindClusters’ function was used to identify clusters. Clusters were identified using the community identification algorithm as implemented in the ‘FindClusters’ function. UMAP dimensionality reduction was computed using the ‘RunUMAP’ function with default parameters.

### 4.12 Identification of DEGs

The ‘FindMarkers’ or ‘FindAllMarkers’ function was applied to identify DEGs with the default parameter ‘test.use = wilcox’. DEGs with an adjusted *p* value < .05 were filtered out.

### 4.13 Functional enrichment analysis

GO enrichment analysis and GSEA were separately conducted to assess the functions of DEGs using the ‘enrichGO’ and ‘GSEA’ functions in the clusterProfiler R package (version 4.2.2).^49^

The ‘AddModuleScore’ function in Seurat was used to score the activity of signalling pathway and the gene sets are listed in Tables S4–S8. EMT signalling pathway activity scores were computed using the R package ‘progeny’.^50^

### 4.14 Developmental trajectory inference

The R package ‘Monocle’ (version 2.22.0)^51^ was adopted to infer the developmental trajectories of neutrophils.

### 4.15 Gene regulatory network analysis

The regulon network was explored using pySCENIC (version 0.12.0),^52^ which analysed the coexpression of transcription factors and their putative target genes.

### 4.16 Cell–cell communication analysis

To infer interaction pairs among subpopulations separately for each sample type and to identify the signalling changes in polyps, we applied the ‘CellChat’ R package (version 1.1.3).^33^ The communication between any two subpopulations was quantified using a communication probability value, and the top 5% probability values of interaction pairs were chosen for subsequent analysis.

### 4.17 Statistical analysis

No statistical methods were used to predetermine the sample size. For scRNA‐seq data, we performed statistical analysis and graph generation using R version 4.1.2 (Foundation for Statistical Computing). For experimental results, unpaired two‐sided *t* tests with equal variance were used, and statistical analysis and graph generation were carried out using GraphPad Prism 8 (GraphPad Software).

## AUTHOR CONTRIBUTIONS

This study was performed in collaboration with all authors. YaD designed and performed the experiments, analysed the data, wrote and revised the manuscript. CL, LH, PX, YaL, YongL, SL, WC, QiangY, YL, QingY, HP, SW, XW, QT, HOuyang, DH, XinL, LL, ZS, JY and XiuL performed the experiments and analysed the data. ZX, YoD and HZ devised the concept, designed the research, supervised the study, wrote and revised the manuscript.

## CONFLICT OF INTEREST STATEMENT

The authors declare no conflict of interest.

## ETHICAL APPROVAL STATEMENT

This study was approved by the Ethics Committee of Hunan Children's Hospital. The ethics committee approval code was HCHLL‐2020‐61. All animal procedures and protocols were approved by the Animal Ethics Committee of Hunan Children's Hospital and followed the guidelines of the Institutional Animal Care and Use Committees of Hunan Children's Hospital. The ethics committee approval code was HCHDWLL‐2020‐03.

## Supporting information

Supporting Information

Supporting Information

## Data Availability

The gene expression data can be obtained from the NCBI GEO database with accession number GEO: GSE244542. Raw sequence data are not public and are protected by controlled access for patient privacy. The code that supports the findings of this study is available from the corresponding authors upon request.
