# Supplementary material for: Single‐cell landscape of the cellular microenvironment in three different colonic polyp subtypes in children
Source: Clin Transl Med. 2024 Jan 24;14(1):e1535. doi: 10.1002/ctm2.1535 (PMC10807352; doi:10.1002/ctm2.1535)
Supplement: Supplementary file 1 — Supporting Information [file CTM2-14-e1535-s002.docx]

**Supplemental Figures**


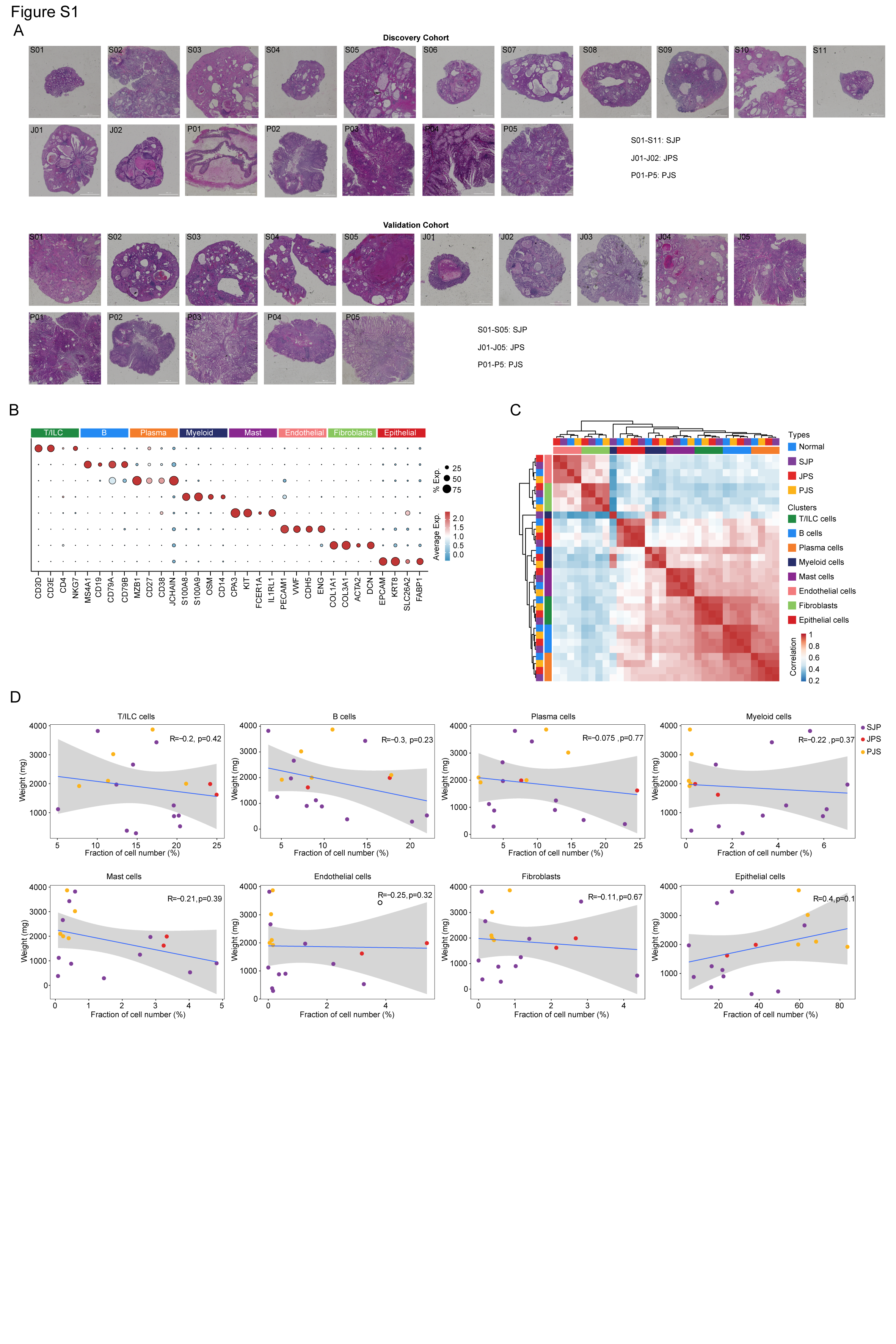


**FIGURE S1.** HE staining of polyps and quality controls related to scRNA-seq analysis. **(**A) Hematoxylin and eosin (HE) staining of polyps applied to scRNA-seq data (top) and mIHC (bottom) in this study. (B) Dot plot displaying the average gene expression of selected marker genes. (C) Grouping of scRNA-seq cell subsets by similarity. (D) Correlation of frequencies of these cell subsets with polyp weights. Each dot represents the cell frequency of each patient with polyp weight, and color denotes different polyp subtypes. Statistical significance was calculated by Spearman correlation (indicated by R), with the p value shown in each graph.


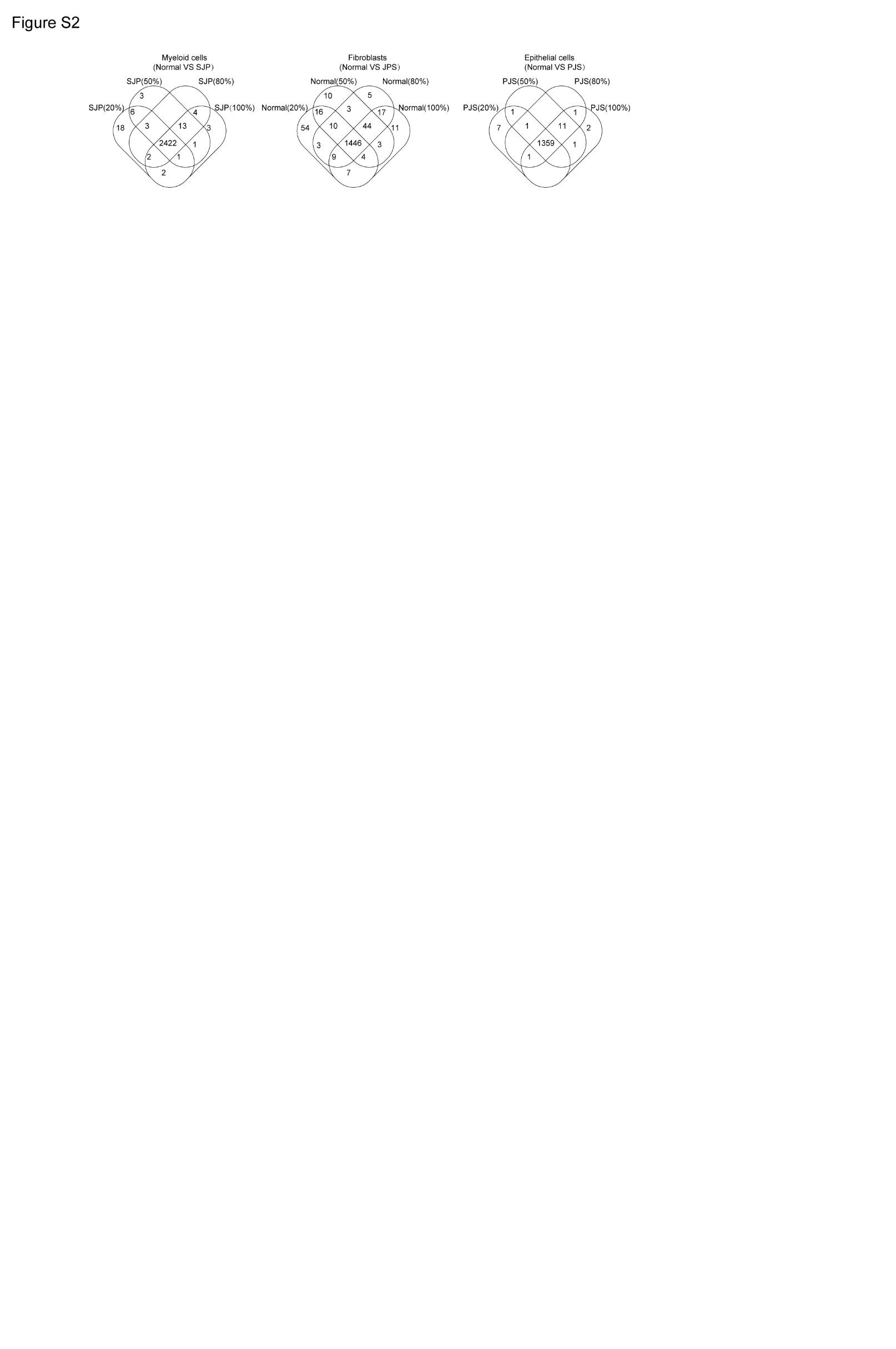


**FIGURE S2.** Venn diagrams of the number of DEGs in myeloid cells (left), fibroblasts (middle) and epithelial cells (right) when we randomly selected 20%, 50%, 80% and 100% of myeloid cells in SJP polyps and then compared them with myeloid cells in nornal tissues, separately. In the same way, we compared different numbers of fibroblasts in normal tissues with fibroblasts in JPS polyps, as well as compared different numbers of epithelial cells in PJS polyps with epithelial cells in normal tissues, separately.


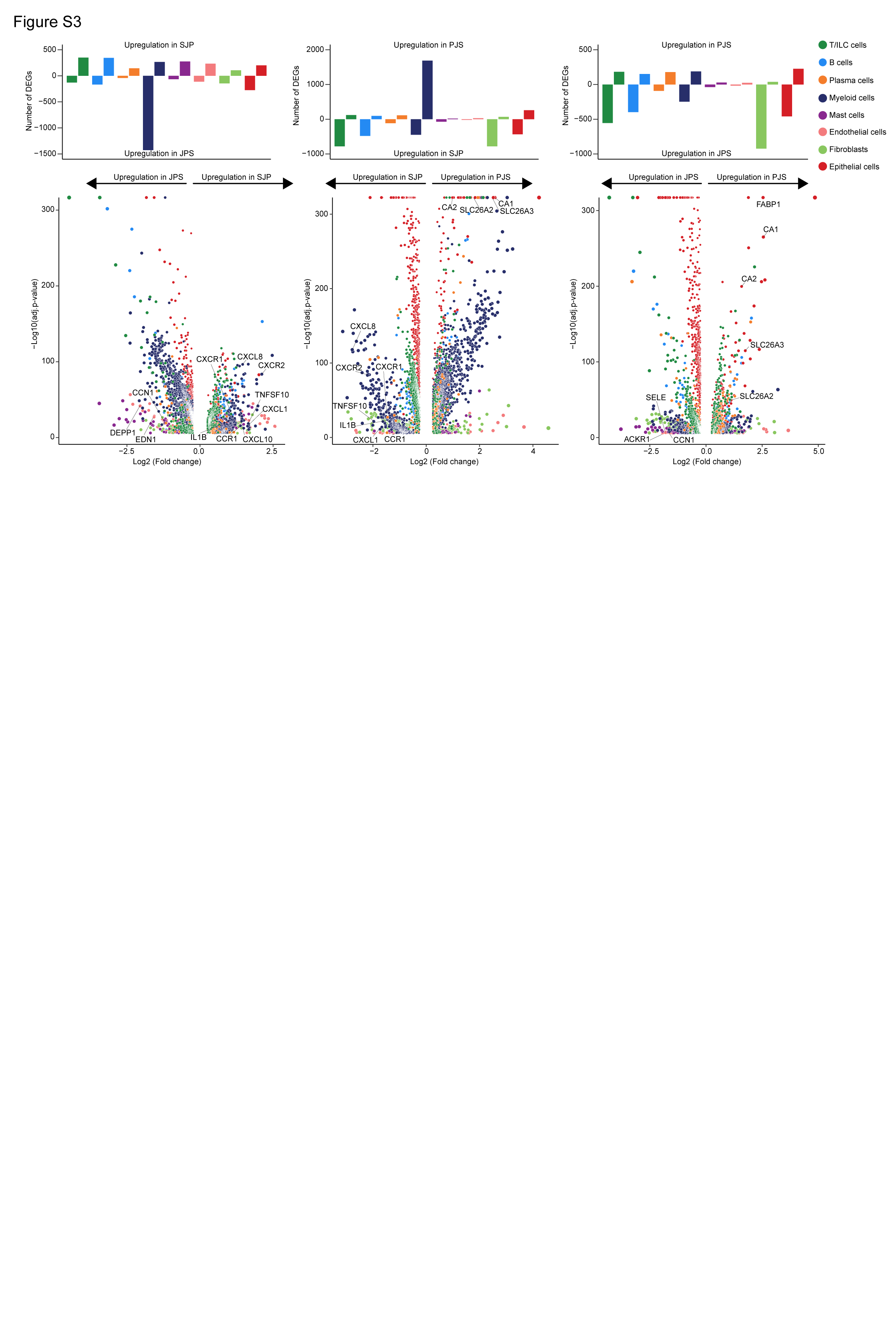


**FIGURE S3.** The DEGs in 8 main cell subsets between different polyp subtypes. Histogram and volcano plot showing the total number (top) and distribution (bottom) of DEGs of each cell subset between different polyp subtypes, respectively.


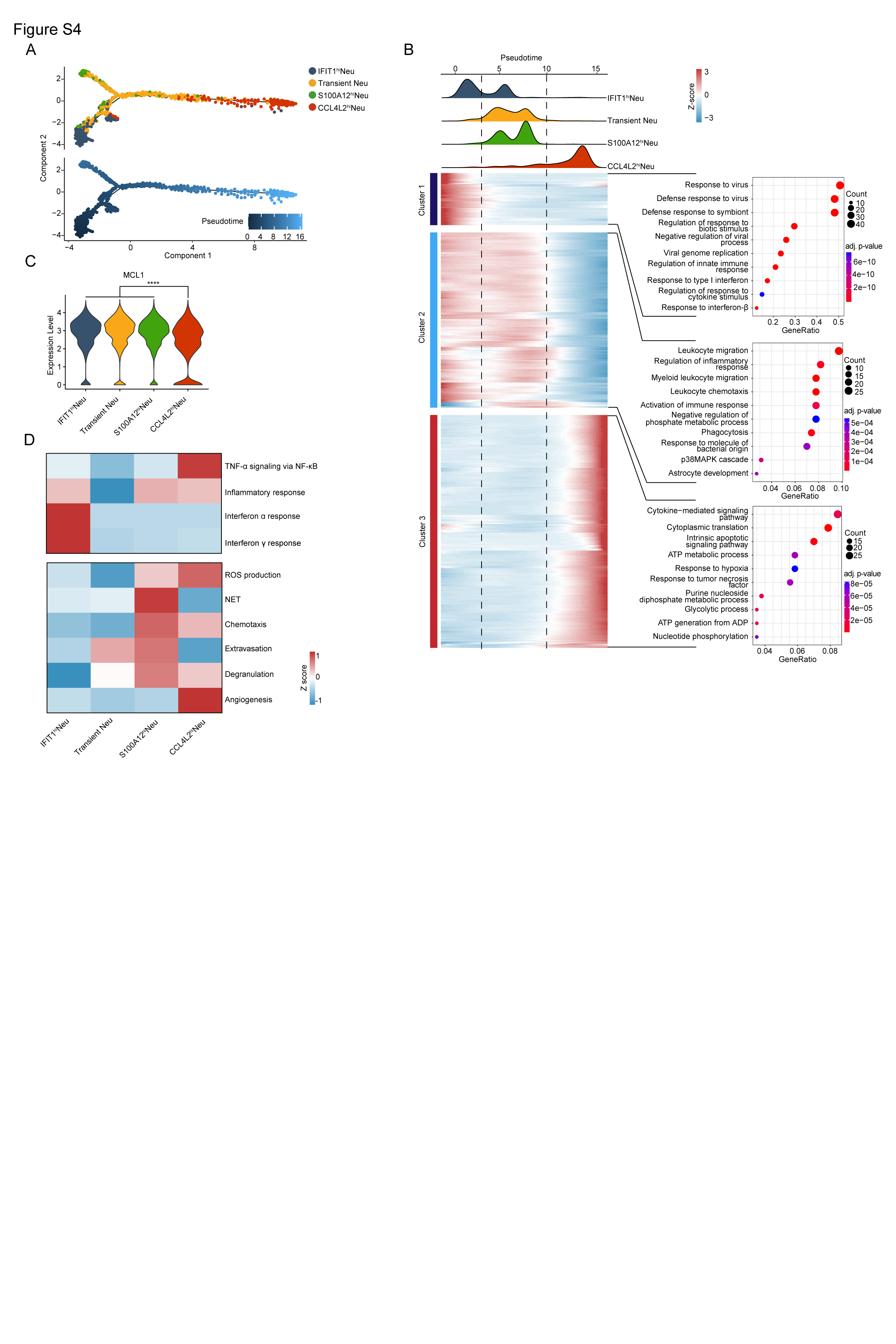


**FIGURE S4.** Developmental trajectory and pathway activities of neutrophil subsets. (A) Monocle 2 trajectory reconstruction analysis of neutrophil differentiation. (B) The cell distribution of each neutrophil subset is shown along pseudotime, color-coded by neutrophil subsets (upper panel). Heatmap showing dynamic expression changes in the top 1,000 highly variable genes (lower panel, left) and related pathways along pseudotime (lower panel, right). (C) Vlnplot showing the expression levels of MCL1 in neutrophil subsets. Statistical analysis was performed using two-sided unpaired Dunn’s (Bonferroni) test; *****p* < 0.0001. (D) Heatmap showing the activities of given pathways in neutrophil subsets.


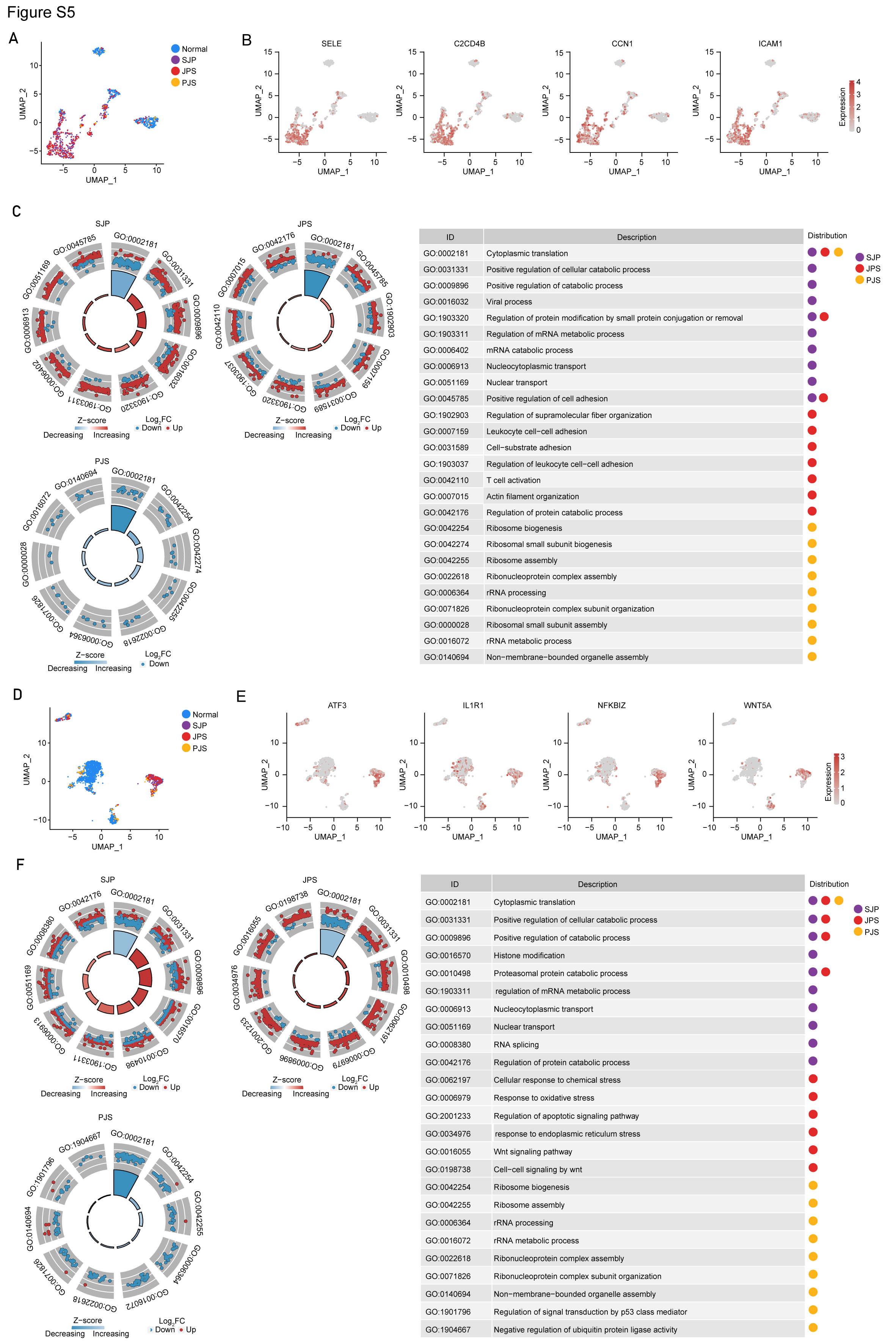


**FIGURE S5.** Endothelial cells and fibroblasts separately show upregulated cell adhesion and EMT signaling in SJP and JPS polyps. (A and D) UMAP plots for endothelial cells (A) and fibroblasts (D) in different sample types. (B and E) Feature plots showing the expression of the indicated genes in endothelial cells (B) and fibroblasts (E). (C and F) Circle plots showing the top 10 GO-enriched pathways in endothelial cells (C) and fibroblasts (F) in SJP, JPS, and PJS polyps (left). The inner ring is a bar plot where the height of the bar indicates the significance of the term (-log_10_ (adj. p value)), and the color corresponds to the z score. The outer ring displays scatter plots of the expression levels (Log_2_FC) of the genes in each term. The table consists of the ID and description of pathways, and the dot indicates the distribution of pathways among three different polyp subtypes (right).


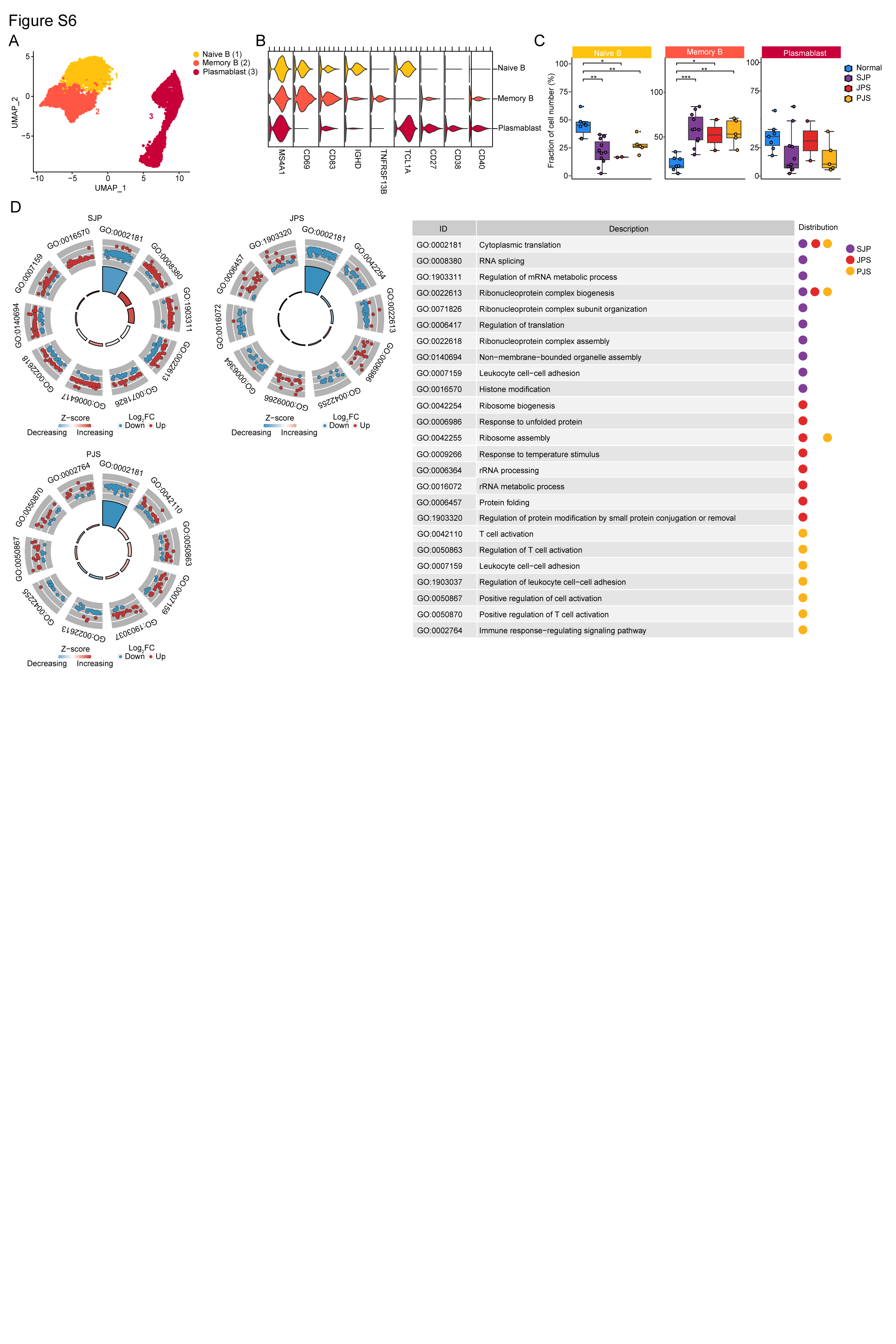


**FIGURE S6.** Memory B cells are increased across different polyp subtypes in pediatric patients. (A) UMAP plot displaying B cells separated into 3 subsets in different sample types. (B) Vlnplot showing subset-specific marker genes. (C) Boxplots comparing the fractions of B-cell subsets in different sample types. Statistical analysis was performed using the Kruskal‒Wallis test; **p* < 0.05, ***p* < 0.01, ****p* < 0.001. (D) Circle plots showing the top 10 GO-enriched pathways in memory B cells in SJP, JPS, and PJS polyps (left). The inner ring is a bar plot where the height of the bar indicates the significance of the term (-log_10_ (adj. p value)), and the color corresponds to the z score. The outer ring displays scatter plots of the expression levels (Log_2_FC) of the genes in each term. The table consists of the ID and description of pathways, and the dot indicates the distribution of pathways among three different polyp subtypes (right).


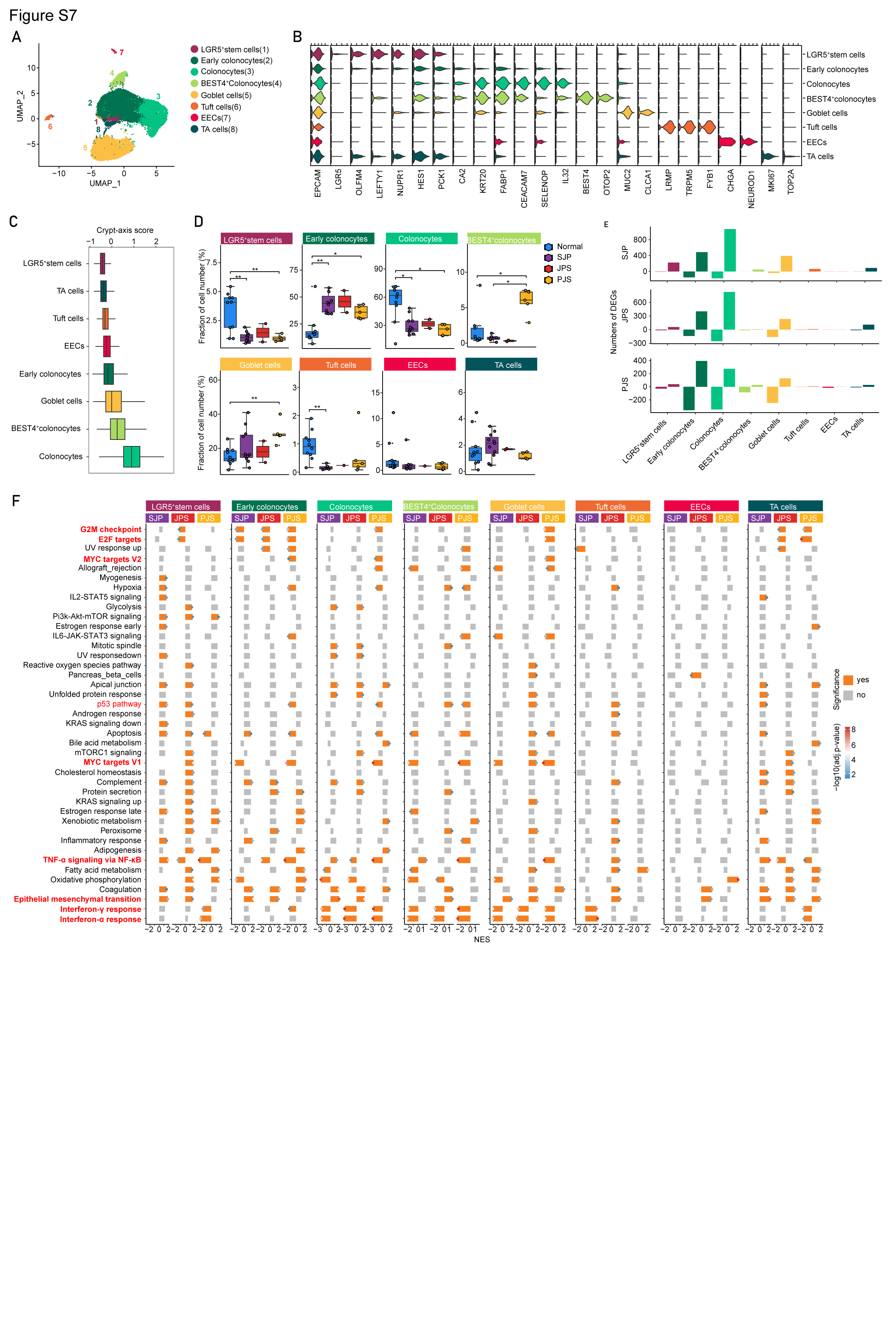


**FIGURE S7.** Subsets of epithelial cells in normal tissues and different polyp subtypes. (A) UMAP plot displaying epithelial cells separated into 8 subsets in different sample types. (B) Heatmap showing subset-specific marker genes. (C) Box and whisker plots of crypt-axis scores generated from the expression of 15 crypt-axis markers. (D) Boxplots comparing the fractions of epithelial subsets in different sample types. Statistical analysis was performed using the Kruskal‒Wallis test; **p* < 0.05, ***p* < 0.01, ****p* < 0.001. (E) Boxplots showing the number of DEGs in each epithelial cell subset across different polyp subtypes. (F) Boxplots showing the activities of hallmark gene sets in epithelial cell subsets across each polyp type.


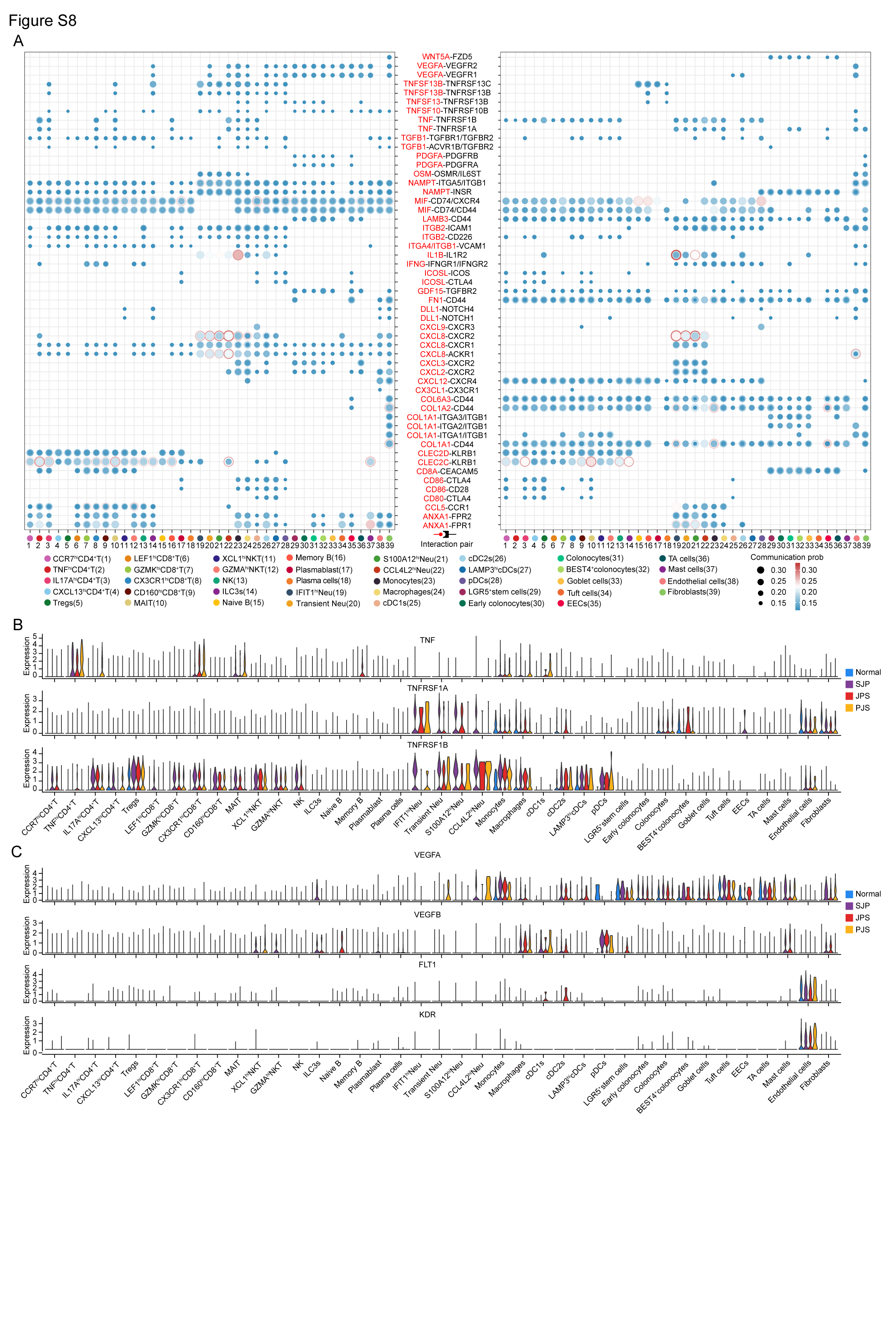


**FIGURE S8.** Cell‒cell interactions in normal tissue and polyps in pediatric patients. (A) Dot plot displaying interaction pairs among cell subsets. Both point size and color indicate the communication probability. (B and C) Vlnplot displaying the expression levels of genes related to TNF (B) and VEGF (C) signaling networks in normal tissue and polyps.


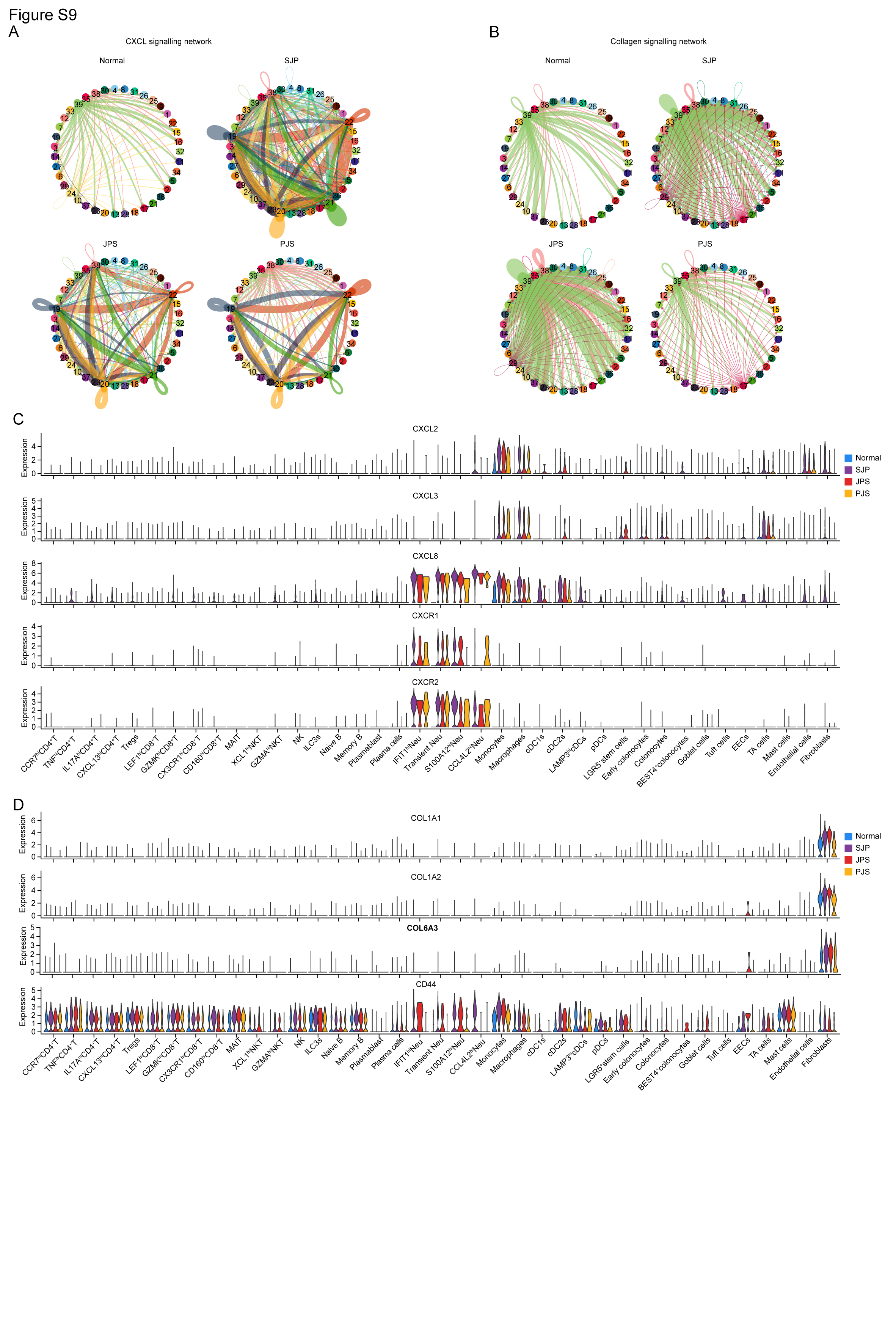


**FIGURE S9.** Enhanced CXCL and collagen signals in polyps in pediatric patients. (A and B) Circle plot displaying the interactions of the CXCL (A) and collagen (B) signal networks among cell subsets across normal tissue and different polyp subtypes. (C and D) Vlnplot displaying the expression levels of genes related to CXCL (C) and collagen (D) signaling networks in normal tissue and polyps.
